# Physician Antipsychotic Overprescribing Letters and Cognitive, Behavioral, and Physical Health Outcomes Among People With Dementia: A Secondary Analysis of a Randomized Clinical Trial

**DOI:** 10.1001/jamanetworkopen.2024.7604

**Published:** 2024-04-25

**Authors:** Michelle Harnisch, Michael L. Barnett, Stephen Coussens, Kali S. Thomas, Mark Olfson, Kiros Berhane, Adam Sacarny

**Affiliations:** 1 PhD Economics Programme, Department of Economics, London School of Economics and Political Science, London, United Kingdom; 2 Department of Health Policy and Management, Harvard T. H. Chan School of Public Health, Boston, Massachusetts; 3 Department of Health Policy and Management, Mailman School of Public Health, Columbia University, New York, New York; 4 Center for Equity in Aging, Johns Hopkins University School of Nursing, Baltimore, Maryland; 5 Department of Psychiatry, Department of Epidemiology, Columbia University Irving Medical Center, New York, New York; 6 Department of Biostatistics, Mailman School of Public Health, Columbia University, New York, New York

## Abstract

**Importance:**

Antipsychotics, such as quetiapine, are frequently prescribed to people with dementia to address behavioral symptoms but can also cause harm in this population.

**Objective:**

To determine whether warning letters to high prescribers of quetiapine can successfully reduce its use among patients with dementia and to investigate the impacts on patients’ health outcomes.

**Design, Setting, and Participants:**

This is a secondary analysis of a randomized clinical trial of overprescribing letters that began in April 2015 and included the highest-volume primary care physician (PCP) prescribers of quetiapine in original Medicare. Outcomes of patients with dementia were analyzed in repeated 90-day cross-sections through December 2018. Analyses were conducted from September 2021 to February 2024.

**Interventions:**

PCPs were randomized to a placebo letter or 3 overprescribing warning letters stating that their prescribing of quetiapine was high and under review by Medicare.

**Main Outcomes and Measures:**

The primary outcome of this analysis was patients’ total quetiapine use in days per 90-day period (the original trial primary outcome was total quetiapine prescribing by study PCPs). Prespecified secondary outcomes included measures of cognitive function and behavioral symptoms from nursing home assessments, indicators of depression from screening questionnaires in assessments and diagnoses in claims, metabolic diagnoses derived from assessments and claims, indicators of use of the hospital and other health care services, and death. Outcomes were analyzed separately for patients living in nursing homes and in the community.

**Results:**

Of the 5055 study PCPs, 2528 were randomized to the placebo letter, and 2527 were randomized to the 3 warning letters. A total of 84 881 patients with dementia living in nursing homes and 261 288 community-dwelling patients with dementia were attributed to these PCPs. There were 92 874 baseline patients (mean [SD] age, 81.5 [10.5] years; 64 242 female [69.2%]). The intervention reduced quetiapine use among both nursing home patients (adjusted difference, –0.7 days; 95% CI, −1.3 to −0.1 days; *P* = .02) and community-dwelling patients (adjusted difference, −1.5 days; 95% CI, −1.8 to −1.1 days; *P* < .001). There were no detected adverse effects on cognitive function (cognitive function scale adjusted difference, 0.01; 95% CI, −0.01 to 0.03; *P* = .19), behavioral symptoms (agitated or reactive behavior adjusted difference, −0.2%; 95% CI −1.2% to 0.8% percentage points; *P* = .72), depression, metabolic diagnoses, or more severe outcomes, including hospitalization and death.

**Conclusions and Relevance:**

This study found that overprescribing warning letters to PCPs safely reduced quetiapine prescribing to their patients with dementia. This intervention and others like it may be useful for future efforts to promote guideline-concordant care.

**Trial Registration:**

ClinicalTrials.gov Identifier: NCT05172687

## Key Points

**Question:**

Did overprescribing warning letters to high-volume primary care physician prescribers of the antipsychotic quetiapine reduce quetiapine use by their patients with dementia without harming patient health outcomes?

**Findings:**

In this secondary analysis of a randomized clinical trial, overprescribing warning letters significantly reduced quetiapine use among patients with dementia living in nursing homes and in the community. There were no detected adverse effects on indicators of cognitive, behavioral, and physical health.

**Meaning:**

This study found that warning letters informed by behavioral science can safely reduce overprescribing to patients with dementia, and related interventions may be broadly useful in promoting guideline-concordant care.

## Teaser

This secondary analysis of a randomized clinical trial examines whether warning letters to high prescribers of quetiapine are associated with reduced prescribing to patients with dementia and investigates the impacts on patients’ health outcomes.

## Introduction

Off-label use of antipsychotics in patients with dementia is commonplace. Approximately 1 in 7 nursing home residents receives an antipsychotic every quarter, and a similar share of people with dementia who live in the community receive an antipsychotic annually.^1,2,3^ Rates of antipsychotic use in older adults with dementia have declined but prescribing persists at high levels.^4^ Antipsychotics are frequently prescribed off-label to treat behavioral symptoms of dementia such as agitation and aggression. Although there is some evidence supporting this use, it can also cause substantial harm.^5,6^ Studies have linked antipsychotic use among people with dementia to increased risks of weight gain, cognitive decline, falls and other injuries, cerebrovascular events, and mortality.^7,8,9,10^

Specialty societies and regulators have, therefore, promoted more judicious use of antipsychotics in dementia care.^11,12,13,14,15^ However, studies on reducing prescribing largely consist of small trials or observational analyses, and evidence from large-scale randomized studies remains limited.^16,17,18,19^ In practice, efforts to further reduce antipsychotic prescribing might achieve modest benefit or could even harm patients. For instance, ongoing antipsychotic use may occur in settings that lack the staffing and other resources to provide effective alternative interventions to ensure patient safety.^20,21^

We seek to fill this gap in empirical evidence through a secondary analysis of a large randomized letter trial that focused on the antipsychotic quetiapine. Quetiapine is the most-prescribed antipsychotic in the US and is frequently used among patients with dementia.^22,23^ The trial enrolled approximately the top 5% of primary care physician (PCP) prescribers of quetiapine in Medicare. A random half of these PCPs were sent a series of overprescribing warning letters about their prescribing of quetiapine. The primary evaluation of this trial found that the letters substantially reduced quetiapine prescribing for at least 2 years.^24^ However, that evaluation studied a considerably smaller patient sample and did not examine health outcomes beyond hospital visits. The current analysis uses linkages to claims and nursing home assessments to evaluate the effects of the letters on health outcomes among these PCPs’ patients with dementia.

In this analysis, we followed patients with dementia over multiple years and evaluated effects on several behavioral, cognitive, metabolic, and other physical health outcomes. These end points align with the adverse effects of quetiapine and other antipsychotics like weight gain that might improve with deprescribing, as well as the potential benefits of these medications like reduced agitation that could deteriorate with indiscriminate deprescribing.

## Methods

### Trial Design

The design of the randomized clinical trial has been described elsewhere,^24^ and we review it briefly here (the trial protocol is also shown in Supplement 1). The Centers for Medicare & Medicaid Services identified the highest-volume PCP prescribers of quetiapine in Medicare. They were allocated (1:1 ratio) to treatment and control groups using a random sequence of numbers. The Centers for Medicare & Medicaid Services sent treatment group PCPs a series of 3 overprescribing warning letters stating that their quetiapine prescribing was high relative to their peers and was under review by Medicare. The protocol for the current secondary analysis (Supplement 1) provides a reproduction of a sample letter. Control PCPs were sent a placebo letter and clarification letter about an unrelated regulation. Initial letter mailings to both groups occurred in April 2015. Follow-up treatment letters were sent in August and October 2015. The original evaluation followed the PCPs and a cohort of patients for 2 years, until April 2017.

This secondary evaluation analyzes patients’ outcomes through December 2018. It was approved by the institutional review boards of Columbia University (New York, New York) and the National Bureau of Economic Research (Cambridge, Massachusetts) as research exempt from informed consent requirements because the data were deidentified, in accordance with 45 CFR §46. The investigators prespecified the analysis plan for the nursing home sample with data blinded to the treatment status of PCPs and publicly archived it in January 2022 before unblinding. They followed the same process for the community-dwelling sample and archived the plan in August 2022.^25^ The current analyses were conducted from September 2021 to February 2024. This study follows the Consolidated Standards of Reporting Trials (CONSORT) reporting guideline.

### Data Sources

We used 2014 to 2018 Medicare fee-for-service claims, Part D prescribing, Minimum Data Set nursing home assessment, and Medicare enrollment data. The data contain records for 100% of study PCPs’ fee-for-service Medicare patients with dementia during this period.

### Study Sample

Our analytic samples consist of patients with dementia receiving care from the study PCPs between 2014 and 2018 residing in nursing homes (nursing home sample) and in the community (community-dwelling sample). In each sample, we assembled repeated cross-sections of patients during 90-day periods relative to study initiation on April 20, 2015, making the unit of analysis the patient-quarter. For instance, outcomes for the first postintervention cohort were measured from April 20 to July 18, 2015.

To define the set of patients in each cohort, we conducted standard prospective claims-based attribution^26,27^ and matched patients to the clinician (physician or advanced practice practitioner) from whom they received the most outpatient or nursing home–based evaluation and management charges in the 90-day (for nursing home patients) or 180-day (for community-dwelling patients) time window directly preceding each period. For the nursing home sample, we only counted evaluation and management services in nursing facilities and used a 90-day window because we expected more frequent practitioner encounters in this sample. We then selected the patients attributed to study PCPs.

We restricted each cohort to patients in fee-for-service Medicare Parts A and B with Part D coverage during the outcome period and time window used for attribution. To limit to patients with dementia, we required a dementia diagnosis during or before the attribution window in the Medicare Chronic Conditions file. Each cohort, therefore, consists of patients with 1 or more dementia diagnosis codes in claims from approximately the previous 3 years.

Nursing home patients were those residing in a nursing home or skilled nursing facility on the last day of the window used for attribution. We excluded patients having a Medicare-covered skilled nursing stay on this day because prescribing during these stays is not covered by Part D. Patients who received no nursing home assessments during the outcome period were also excluded because we could not measure their nursing home–specific outcomes. Community patients were those who did not reside in a nursing facility on the last day of the attribution window. By construction, the same patient could only enter both samples in different quarters.

### Patient Outcomes

The primary end point was the days of quetiapine received in the 90-day outcome period, as measured by the patient’s prescription drug fills. This end point captures quetiapine treatment initiations and changes in receipt among continuing patients. We also analyzed a variety of claims-based secondary outcomes to measure changes in patients’ health and health care use. These outcomes include depression and metabolic diagnoses, health care encounters, and death. Diagnosis outcomes were defined as the patient having at least 1 inpatient, outpatient, skilled nursing, or professional claim with a relevant diagnosis code.

For patients in nursing homes, we constructed additional health indicators from the assessment data, such as measures of patients’ cognitive function (Cognitive Function Scale),^28^ behavioral symptoms (Agitated and Reactive Behavior Scale),^29^ and depression screening (PHQ-9; Patient Health Questionnaire–9).^30^ These scales have been extensively validated and provide a single overall measurement of each outcome domain. We defined a positive depression screen as a PHQ-9 score of 10 or higher.^31,32^

We further analyzed alternative measures of quetiapine use, such as prescriptions by the patient’s attributed PCP, any prescriptions, and milligrams prescribed. We also studied potential patterns of substitution toward other antipsychotics, including first-generation and second-generation agents, and other psychoactive medication classes frequently used in dementia management, specifically antidepressants, benzodiazepines, gabapentinoids, mood stabilizers, and other sedative-hypnotics.

### Statistical Analysis

To estimate the effect of letters on the primary and secondary end points, we used multivariable regression. The study took a modified intention-to-treat approach by analyzing patients of study PCPs according to methods described in the Study Sample subsection. We adjusted for period, patient-level and prescriber-level end points as measured at baseline, and patient-level demographic characteristics to increase the precision of estimates.^33^ All models used robust variance techniques by clustering SEs at the level of the attributed PCP (the level of randomization).^34^ Two-sided hypothesis tests with *P* < .05 were considered significant. Secondary end points were treated as exploratory and were not adjusted for multiple comparisons.

We conducted exploratory subgroup analyses by patient race and ethnicity, age, sex, and Medicare-Medicaid dual eligibility status. Race and ethnicity were extracted from Medicare enrollment data to assess potential inequities in the effects of the letters. We also divided the nursing home sample according to 3 facility indicators: the share of long-stay residents receiving an antipsychotic, the star rating for safety inspection deficiencies, and the star rating for staffing. Data were analyzed using Stata/MP statistical software version 17.0 (StataCorp).

## Results

Of the 5055 prescribers in the original trial, 2528 were randomized to the placebo letter, and 2527 were randomized to the 3 warning letters. Among those in the trial, 1885 prescribers had nursing home patients, and 4584 had community-dwelling patients, with some having patients in both settings (eFigure 1 in Supplement 2). A total of 84 881 patients living in nursing homes and 261 288 community-dwelling patients were attributed to study prescribers. There were 92 874 baseline patients (mean [SD] age, 81.5 [10.5] years; 64 242 female [69.2%]). Among 22 333 baseline nursing home patients, the mean (SD) age was 82.9 (10.5) years, 16 233 (72.7%) were female, 3526 (15.8%) were Black, 1289 (5.8%) were Hispanic, 16 898 (75.7%) were White, and 620 (2.8%) were of other races (American Indian/Alaska Native, Asian/Pacific Islander, other, and unknown). Among the 70 541 baseline community-dwelling patients, the mean [SD] age was 81.1 (10.5) years, 48 009 (68.1%) were female, 7409 (10.5%) were Black, 9669 (13.7%) were Hispanic, 49 495 (70.2%) were White, and 3968 (5.6%) were of other races. Baseline patients in both groups were frequently prescribed quetiapine: those in nursing homes received a mean (SD) of 12.3 (36.7) days of quetiapine per quarter whereas those in the community received 10.3 (31.3) days (Table 1 and Table 2). In the nursing home sample, study PCPs had a mean (SD) of 11.7 (18.6) dementia patients in nursing homes and prescribed quetiapine to 1.5 of them at baseline. PCPs in the community sample had a mean (SD) of 15.4 (19.6) community-dwelling patients at baseline and prescribed quetiapine to 1.8 patients.

**Table 1.** Baseline Characteristics of Study Patients With Dementia

Characteristic	Patients, No. (%) (N = 92 874)			
	Nursing home sample		Community-dwelling sample	
	Control (n = 11 925)	Treatment (n = 10 408)	Control (n = 36 605)	Treatment (n = 33 936)
Quetiapine receipt, mean (SD)				
Days	12.0 (36.5)	12.7 (36.9)	10.4 (31.5)	10.2 (31.1)
Days from attributed primary care physician	10.3 (33.9)	10.8 (34.1)	7.2 (26.0)	7.0 (25.6)
Any receipt	1478 (12.4)	1367 (13.1)	4392 (12.0)	4027 (11.9)
Milligrams, mean (SD)	1225 (5809)	1356 (6061)	1106 (5390)	1161 (5672)
Age, mean (SD), y	82.8 (10.5)	82.9 (10.5)	81.3 (10.5)	80.9 (10.5)
Sex				
Female	8656 (72.6)	7577 (72.8)	25 070 (68.5)	22 939 (67.6)
Male	3269 (27.4)	2831 (27.2)	11 535 (31.5)	10 997 (32.4)
Race and ethnicity				
Black	1897 (15.9)	1629 (15.7)	3812 (10.4)	3597 (10.6)
Hispanic	715 (6.0)	574 (5.5)	4662 (12.7)	5007 (14.8)
White	8997 (75.4)	7901 (75.9)	25 999 (71.0)	23 496 (69.2)
Other^a^	316 (2.6)	304 (2.9)	2132 (5.8)	1836 (5.4)
Chronic conditions, mean (SD), No.	5.0 (2.3)	5.0 (2.3)	5.0 (2.5)	5.0 (2.5)
Time since first dementia diagnosis, mean (SD), y	5.4 (3.9)	5.4 (3.9)	4.0 (3.6)	3.9 (3.5)
Days in nursing home	705.7 (841.3)	701.2 (816.4)	Not applicable	Not applicable
Dual Medicare-Medicaid eligible	8089 (67.8)	7081 (68.0)	15 217 (41.6)	14 841 (43.7)

^a^ Includes patients with a race code of American Indian/Alaska Native, Asian/Pacific Islander, other, and unknown.

**Table 2.** Characteristics of Study PCPs

Characteristic	PCPs, No. (%) (N = 5055)^a^			
	Nursing home sample		Community-dwelling sample	
	Control (n = 958)	Treatment (n = 927)	Control (n = 2303)	Treatment (n = 2281)
Primary specialization^b^				
General practitioner	23 (2.4)	23 (2.5)	84 (3.6)	99 (4.3)
Family medicine	435 (45.4)	427 (46.1)	1058 (45.9)	1098 (48.1)
Internal medicine	500 (52.2)	477 (51.5)	1161 (50.4)	1084 (47.5)
Any specialization code for geriatrics	80 (8.4)	96 (10.4)	147 (6.4)	154 (6.8)
Sex				
Female	128 (13.4)	122 (13.2)	389 (16.9)	393 (17.2)
Male	830 (86.6)	805 (86.8)	1914 (83.1)	1888 (82.8)
Patients with dementia at baseline, mean (SD), No.^c^				
Total	12.3 (19.2)	11.1 (18.0)	15.9 (21.4)	14.9 (17.7)
Prescribed quetiapine	1.5 (2.7)	1.5 (2.8)	1.9 (3.5)	1.8 (3.3)
Prescribed any antipsychotics	3.3 (5.7)	3.0 (5.4)	3.4 (5.7)	3.1 (5.4)

Abbreviation: PCP, primary care physician.

^a^ Among these PCPs, 1853 treated patients living in both settings and 32 did not treat any patients in either setting.

^b^ A small number of practitioners (<11) in the community-dwelling sample later changed their specialization to a field outside primary care. We classify them here by the primary care specialization that triggered their entry into the study.

^c^ Number of patients at baseline attributed to study PCP. Refers to nursing home patients or community-dwelling patients as given by the column heading.

### Prescribing Outcomes

Across all postintervention periods, the mean (SD) days of quetiapine received by nursing home patients was 10.3 (34.2) in the control group and 10.1 (33.6) in the treatment group. Letters reduced quetiapine receipt by 0.7 days (adjusted difference; 95% CI, −1.3 to −0.1 days; *P* = .02), or 6.7% of the control mean (Table 3). Among community-dwelling patients, the mean (SD) days of quetiapine receipt was 9.9 (31.4) in the control group and 8.2 (28.6) in the treatment group. In this sample, letters reduced quetiapine receipt by 1.5 days (adjusted difference; 95% CI, −1.8 to −1.1 days; *P* < .001), or 14.8% of the control mean.

**Table 3.** Effect of the Intervention on Primary and Secondary Outcomes^a^

Variable	Nursing home patients			Community-dwelling patients		
	Control group, No. (%)^b^	Adjusted difference, d (95% CI)	*P* value	Control group, No. (%)^b^	Adjusted difference, d (95% CI)	*P* value
Prescribing						
Quetiapine receipt						
Days, mean (SD)	10.3 (34.2)	−0.7 (−1.3 to −0.1)	.02	9.9 (31.4)	−1.5 (−1.8 to −1.1)	<.001
Days from attributed primary care physician, mean (SD)	8.6 (31.1)	−0.7 (−1.3 to −0.2)	.01	6.6 (25.2)	−1.3 (−1.5 to −1.0)	<.001
Any receipt	16 647 (10.4)	−0.6 (−1.2 to −0.1)	.03	54 871 (11.0)	−1.6 (−1.9 to −1.3)	<.001
Milligrams, mean (SD)	1057 (5362)	−64 (−139 to 12)	.10	1024 (5112)	−121 (−161 to −81)	<.001
Receipt of other antipsychotics						
Days, mean (SD)	13.0 (37.0)	0.4 (−0.2 to 0.9)	.24	8.2 (29.3)	0.1 (−0.2 to 0.3)	.58
Any receipt	22 404 (14.0)	0.4 (−0.2 to 1.0)	.17	45 800 (9.2)	0.2 (−0.1 to 0.5)	.15
Received other psychiatric medication	103 646 (64.9)	−0.2 (−1.1 to 0.6)	.59	269 974 (54.1)	0.0 (−0.4 to 0.5)	.91
Cognitive and behavioral health						
Cognitive function scale score, mean (SD)^c^	2.58 (0.99)	0.01 (−0.01 to 0.03)	.19	NA	NA	NA
Agitated or reactive behavior	29 570 (18.6)	−0.2 (−1.2 to 0.8)	.72	NA	NA	NA
Depression screen positive^d^	8340 (5.3)	−0.5 (−1.2 to 0.2)	.13	NA	NA	NA
Depression diagnosis	48 859 (30.6)	−1.3 (−2.6 to 0.1)	.07	85 955 (17.2)	−0.5 (−1.1 to 0.1)	.07
Metabolic indicators and diagnoses						
Weight loss	12 422 (7.8)	0.3 (−0.0 to 0.7)	.06	NA	NA	NA
Body mass index, mean (SD)^e^	26.4 (6.5)	−0.0 (−0.1 to 0.1)	.57	NA	NA	NA
Diabetes diagnosis	55 499 (34.8)	−0.1 (−0.9 to 0.6)	.70	158 309 (31.7)	0.1 (−0.4 to 0.6)	.70
Hyperlipidemia diagnosis	45 481 (28.5)	−0.1 (−1.5 to 1.4)	.94	193 232 (38.7)	0.6 (−0.2 to 1.4)	.15
Hypertension diagnosis	111 049 (69.5)	−0.0 (−1.5 to 1.4)	.95	316 600 (63.5)	−0.1 (−0.7 to 0.6)	.83
Hyperglycemia diagnosis	2693 (1.7)	−0.1 (−0.3 to 0.1)	.53	18 522 (3.7)	0.2 (−0.2 to 0.7)	.32
Other indicators of adverse events						
Death	6833 (4.3)	0.0 (−0.1 to 0.2)	.65	20 834 (4.2)	−0.1 (−0.3 to −0.0)	.04
Any emergency department visit	17 087 (10.7)	0.3 (−0.3 to 0.8)	.31	79 238 (15.9)	0.1 (−0.2 to 0.4)	.54
Any inpatient stay	17 889 (11.2)	−0.3 (−0.7 to 0.1)	.19	66 331 (13.3)	0.0 (−0.3 to 0.3)	.81
Entry to skilled nursing facility or nursing facility	NA	NA	NA	22 607 (4.5)	−0.0 (−0.2 to 0.1)	.68
Any use of restraints	2660 (1.7)	−0.0 (−0.3 to 0.3)	.92	NA	NA	NA

Abbreviation: NA, not applicable.

^a^ All outcomes were measured within 90-day periods and were based on claims and assessments with dates of service or target dates during the period. The number of observations is 299 729 patient-periods (84 881 distinct patients) for the nursing home sample and 965 510 patient-periods (261 288 distinct patients) for the community-dwelling sample. For the nursing home sample, assessment outcomes are occasionally missing and effective sample sizes may be slightly smaller (minimum number of observations 290 861).

^b^ Control group number of observations in patient-periods is provided for binary outcomes.

^c^ Measured by the Cognitive Function Scale, ranging from 1 (cognitively intact) to 4 (severely impaired).

^d^ Defined as a Patient Health Questionnaire–9 score of 10 or higher.

^e^ Body mass index is calculated as weight in kilograms divided by height in meters squared.

**Figure 1.**
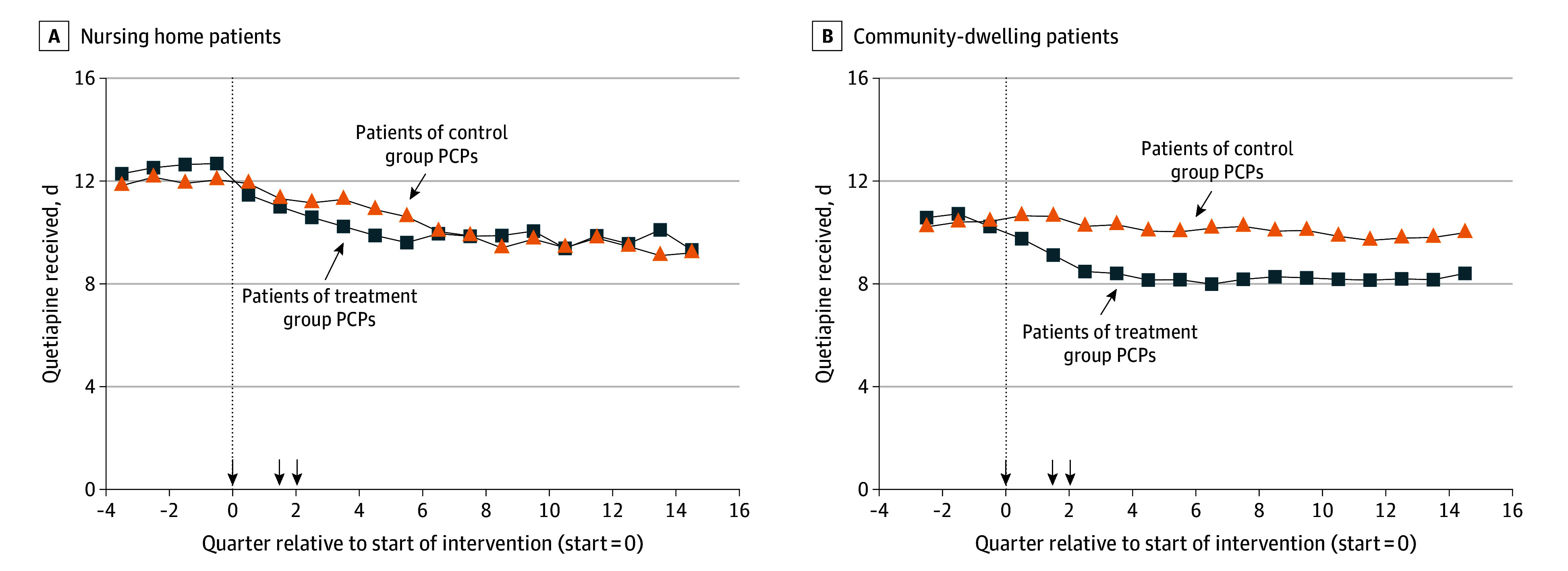
Time Series of Quetiapine Receipt for Treatment and Control Patients Each point represents the mean days of quetiapine received by patients of treatment and control group study primary care physicians (PCPs) during 90-day periods relative to study initiation in April 2015. The number of observations within each group and quarter ranges between 8658 and 13 114 patients for the nursing home sample (A) and between 29 347 and 39 506 patients for the community sample (B). Patients with multiple study PCPs across periods are included in the time series. Arrowheads on x-axis denote when warning letters were sent to treatment group PCPs.

Among nursing home patients, reductions in quetiapine receipt were concentrated in the first 6 quarters of the study, whereas reductions persisted for the entire 15 quarter postintervention period among patients in the community (Figure 1). Restricting the analysis of nursing home patients to the first 6 quarters, letters reduced quetiapine use by 1.1 days per quarter (adjusted difference; 95% CI, −1.6 to −0.5 days; *P* < .001; control mean, 11.2 days; treatment mean, 10.5 days) (eTable 1 in Supplement 2).

Relative effects were similar using other measures of quetiapine use. In both samples, there was no detected effect on use of other antipsychotics. There were no significant changes in receipt of other psychoactive medications commonly used in dementia management, including those used off-label to manage behavioral symptoms like antidepressants^35^ and mood stabilizers^23^ (eTable 2 in Supplement 2).

### Health Outcomes

#### Nursing Home Patients

Among nursing home patients, there were no statistically significant adverse changes in cognitive or behavioral health measures coincident with the decline in quetiapine use. Specifically, there was no detected effect on cognitive function (mean [SD] score, 2.6 [0.99] for control vs 2.6 [0.98] for treatment; adjusted difference, 0.01; 95% CI, −0.01 to 0.03; *P* = .19; scale range 1-4 with higher values indicating more limited function). We also found no detectable effect on the share of patients with any agitated or reactive behavior (control mean, 18.6%; treatment mean, 18.5%; adjusted difference −0.2%; 95% CI, −1.2% to 0.8%; *P* = .72).

Measures of depression rates were similar but generally lower in the treatment group compared with control. Specifically, the share with screen positive depression was not significantly different (control mean, 5.3%; treatment mean, 4.3%; adjusted difference, −0.5%; 95% CI, −1.2% to 0.2%; *P* = .13) nor was share with depression diagnoses in claims (control mean, 30.6%; treatment mean, 28.4%; adjusted difference, −1.3%; 95% CI, −2.6% to 0.1%; *P* = .07). Alternative constructions of cognitive, behavioral, and mental health end points yielded similar results (eTable 3 in Supplement 2).

A higher percentage of treatment patients reported weight loss vs the control group, although the difference was not significant (control mean, 7.8%; treatment mean, 8.1%; adjusted difference, 0.3%; 95% CI, −0.0% to 0.7%; *P* = .06). Rates of metabolic diagnoses in claims were similar in the treatment and control groups. Indicators of more severe adverse outcomes, including emergency department use, inpatient hospital admission, death, and use of restraints, were not significantly different between treatment and control groups. Analyses of further prespecified outcomes also did not suggest harm (eTable 4 in Supplement 2).

#### Community-Dwelling Patients

Results were similar for patients living in the community, although only claims-based health indicators were available for this population. As we observed for patients living in nursing homes, there was a decrease in the share with depression diagnoses that was not statistically significant (control mean, 17.2%; treatment mean, 16.6%; adjusted difference, −0.5%; 95% CI, −1.1% to 0.1%; *P* = .07). There were no detected adverse impacts on more severe health end points, including rates of hospital use or entry to nursing facilities. The risk of death was statistically significantly lower for treated vs control patients (control mean, 4.2%; treatment mean, 3.9%; adjusted difference, −0.1%; 95% CI, −0.3% to −0.0%; *P* = .04).

### Subgroup Analyses

**Figure 2.**
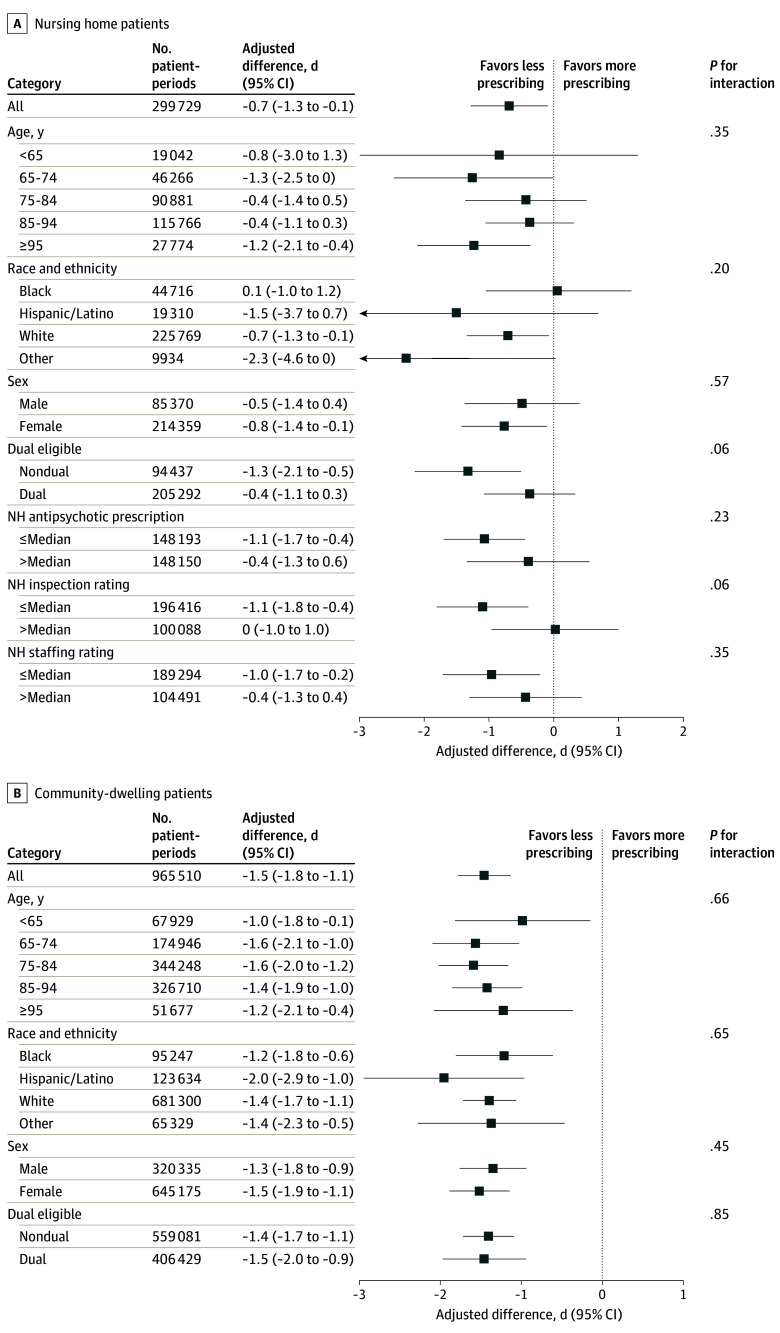
Effect of the Intervention on Quetiapine Receipt in Patient Subgroups Forest plots show effects of the intervention on quetiapine receipt (measured in days) for patient subgroups. See eFigures 2-4 in Supplement 2 for subgroup results on cognitive, behavioral, and physical health end points. The first row shows the effect for the full sample (Table 3). In panel A, the final 3 subgroup analyses divide the sample according to indicators at the patient’s nursing home (NH), including its antipsychotic prescribing rate, its safety inspection star rating, and its staffing rating. Sample size is given in patient-periods. Sample sizes for NH subgroup analyses do not add to total because NH indicators were not available for a small number of facilities. Error bars show 95% CIs. Bars are truncated at the limits of the x-axis scale to improve visualization of effect differences. Truncation is indicated by arrowhead at end of bar. Interaction *P* values are given for the null hypothesis that effects were equal across the subgroups. Other race/ethnicity refers to American Indian/Alaska Native, Asian/Pacific Islander, other, and unknown.

Among nursing home patients, reductions in quetiapine receipt were particularly pronounced among relatively young (aged 65-74 years) and relatively old (aged ≥95 years) individuals (Figure 2A). Although deprescribing was statistically significant for White patients only, the effects were measured imprecisely for other groups owing to smaller subgroup samples. In turn, we fail to reject that the reduction in quetiapine receipt was the same across racial and ethnic groups. Impacts appeared to focus on individuals who were not dually eligible for Medicare and Medicaid, although differences were not clearly distinguishable. The effects were large and statistically significant in nursing homes with poorer star ratings and lower prestudy levels of antipsychotic prescribing.

Among patients living in the community, reductions in quetiapine prescribing were comparatively similar across subgroups (Figure 2B). In this sample, differences on the basis of race, ethnicity, and dual eligibility status were muted compared with the nursing home sample.

There were no consistent signs of harm across key nursing home assessment–based health outcomes (eFigure 2 in Supplement 2) and key claims-based health outcomes (eFigures 3 and 4 in Supplement 2) in the subgroups. In turn, subgroups that experienced more substantial deprescribing, like White and/or non–dually eligible nursing home patients, did not show corresponding deteriorations in these indicators.

## Discussion

This secondary analysis of a randomized clinical trial provides evidence that a low-cost letter intervention informed by behavioral science can reduce prescribing of quetiapine to patients with dementia in nursing home and community settings. Lower levels of quetiapine use were not accompanied by adverse health outcomes, including adverse changes in cognitive function, behavioral symptoms, metabolic diagnoses, and hospitalization. We further found encouraging, although not statistically significant, differences in rates of depression diagnoses in the direction of benefit. There were also signs of decreased risk of death among patients living in the community. Taken together, these findings suggest that the deprescribing induced by the letters was unlikely to harm and may have helped patients.

This evaluation provides new evidence on the value of ongoing interventions to reduce antipsychotic use in dementia care. It addresses several limitations of previous trials^16,17,19^ that had suggested deprescribing could be done safely. The sample size of the present study was 2 to 3 orders of magnitude greater than that of previous trials, greatly increasing statistical power to detect patient harms. Nevertheless, our large-scale study reaffirms the potential for interventions to promote safe deprescribing. This intervention was also conducted when second-generation antipsychotics were dominant, further increasing its relevance for contemporary practice. Finally, the letter approach evaluated here is likely lower-cost than many alternative interventions.

Reductions in quetiapine use were larger and more durable among patients with dementia who lived in the community, pointing to the potential for interventions to promote long-lasting deprescribing. These impacts may reflect the stronger language of these letters compared with other overprescribing letters.^24,36,37^ The effects were smaller and shorter-lived for nursing home patients. It is possible that behavioral symptoms were more severe among patients in nursing homes,^38^ improving the benefit-harm trade-off of antipsychotics. In addition, prescribing in nursing homes may depend on both PCP-level and nursing home–level factors,^21^ diminishing the effect of a PCP-directed intervention. This concern highlights the potential value of coordinating interventions with nursing homes to ensure that their effects endure.

There are longstanding inequities in the quality of nursing and dementia care, particularly by race.^39,40,41^ The subgroup analyses establish that among patients living in the community, this intervention promoted broad deprescribing across racial and ethnic groups. Effects were also similar by dual eligibility status, which can serve as a proxy for income.

Among nursing home patients, the statistical power to detect differences in effects across groups was more limited, and observed differences by race and ethnicity and dual eligibility were more difficult to discern from statistical noise. In the nursing home setting, facility-level effects may also play a role in such differences. Because of power considerations, our analyses of intervention effects by race and dual eligibility status did not distinguish within-nursing home effect heterogeneity and between-nursing home effect heterogeneity.

Encouragingly, the intervention appeared to reduce quetiapine prescribing at nursing homes with poorer preintervention star ratings and did so without signals of harm. Although other studies^20,21^ have suggested that antipsychotics may sometimes be prescribed in lieu of adequate staffing, these results suggest that facilities with less staffing still have opportunities to safely deprescribe. These findings also point to the possibility that the intervention had similar effects across patient groups within a nursing home, but that effects differed between facilities.

We note 2 considerations for the generalizability of this work. One is that health impacts of the intervention may differ for patient groups outside the scope of our study for whom the use of antipsychotics is backed by strong evidence, including patients with serious mental illnesses who often benefit greatly from these medications. Second, the future value of this approach depends on whether physicians would react similarly to ongoing warning letters sent by other stakeholders.

### Limitations

There are several limitations to this study. First, we did not directly observe administration of quetiapine and instead proxied for it with prescription drug fills. Second, we could not observe results for people enrolled in Medicare Advantage. Third, the claims-based and assessment-based outcomes might have been subject to measurement error and, in some cases, underascertainment of diagnoses.^42,43^

## Conclusions

A letter intervention targeting high-volume primary care physician prescribers of quetiapine reduced receipt of this medication among their patients with dementia without detectable adverse health impacts. Our results highlight the value of simple deprescribing interventions for clinicians, specialty societies, and regulators seeking to improve the quality of dementia care. Related interventions could promote guideline-concordant care more broadly.

## Notes

Supplement 1.
Trial Protocol and Statistical Analysis Plan

Supplement 2. **eFigure 1.** CONSORT Flow Diagram of PCPs and Their Patients in Study **eFigure 2.** Effect of the Intervention on Nursing Home Assessment Outcomes in Patient Subgroups **eFigure 3.** Effect of the Intervention on Claims-Based Outcomes in Nursing Home Patient Subgroups **eFigure 4.** Effect of the Intervention on Claims-Based Outcomes in Community-Dwelling Patient Subgroups **eTable 1.** Effect of the Intervention During the First Six Quarters for Nursing Home Patients **eTable 2.** Effect on Other Prescribing Outcomes **eTable 3.** Effect on Alternative Measures of Cognitive, Behavioral, and Mental Health **eTable 4.** Effect on Additional Utilization Outcomes

Supplement 3.
Data Sharing Statement

## References

[1] Centers for Medicare & Medicaid Services. National partnership to improve dementia care in nursing homes: antipsychotic medication use data report. April 2022. Accessed April 12, 2023. https://www.cms.gov/files/document/antipsychotic-medication-use-data-report-2021q4-updated-07292022.pdf

[2] Centers for Medicare & Medicaid Services. Antipsychotic use in Part D enrollees with dementia. November 16, 2015. Accessed March 11, 2024. https://www.cms.gov/Medicare/Prescription-Drug-Coverage/PrescriptionDrugCovGenIn/Downloads/Antipsychotic-Use-in-Part-D-Enrollees-with-Dementia-v12092015.pdf

[3] Government Accountability Office. Antipsychotic drug use: HHS has initiatives to reduce use among older adults in nursing homes, but should expand efforts to other settings. January 2015. Accessed March 11, 2024. https://www.gao.gov/assets/gao-15-211.pdf

[4] Lucas JA, Bowblis JR. CMS strategies to reduce antipsychotic drug use in nursing home patients with dementia show some progress. Health Aff (Millwood). 2017;36(7):1299-1308. doi:10.1377/hlthaff.2016.1439 28679818

[5] Tampi RR, Tampi DJ, Balachandran S, Srinivasan S. Antipsychotic use in dementia: a systematic review of benefits and risks from meta-analyses. Ther Adv Chronic Dis. 2016;7(5):229-245. doi:10.1177/2040622316658463 27583123 PMC4994396

[6] Kales HC, Gitlin LN, Lyketsos CG. Assessment and management of behavioral and psychological symptoms of dementia. BMJ. 2015;350:h369. doi:10.1136/bmj.h369 25731881 PMC4707529

[7] Steinberg M, Lyketsos CG. Atypical antipsychotic use in patients with dementia: managing safety concerns. Am J Psychiatry. 2012;169(9):900-906. doi:10.1176/appi.ajp.2012.12030342 22952071 PMC3516138

[8] Zheng L, Mack WJ, Dagerman KS, . Metabolic changes associated with second-generation antipsychotic use in Alzheimer’s disease patients: the CATIE-AD study. Am J Psychiatry. 2009;166(5):583-590. doi:10.1176/appi.ajp.2008.08081218 19369318 PMC2891018

[9] Maust DT, Kim HM, Seyfried LS, . Antipsychotics, other psychotropics, and the risk of death in patients with dementia: number needed to harm. JAMA Psychiatry. 2015;72(5):438-445. doi:10.1001/jamapsychiatry.2014.3018 25786075 PMC4439579

[10] Vigen CLP, Mack WJ, Keefe RSE, . Cognitive effects of atypical antipsychotic medications in patients with Alzheimer’s disease: outcomes from CATIE-AD. Am J Psychiatry. 2011;168(8):831-839. doi:10.1176/appi.ajp.2011.08121844 21572163 PMC3310182

[11] US Food and Drug Administration. Information for healthcare professionals: conventional antipsychotics. June 16, 2008. Accessed March 13, 2023. https://wayback.archive-it.org/7993/20170722190727/https://www.fda.gov/Drugs/DrugSafety/PostmarketDrugSafetyInformationforPatientsandProviders/ucm124830.htm

[12] The American Geriatrics Society Beers Criteria® Update Expert Panel. American Geriatrics Society 2023 updated AGS Beers Criteria^®^ for potentially inappropriate medication use in older adults. J Am Geriatr Soc. 2023;71(7):2052-2081. doi:10.1111/jgs.18372 37139824 PMC12478568

[13] Reus VI, Fochtmann LJ, Eyler AE, . The American Psychiatric Association practice guideline on the use of antipsychotics to treat agitation or psychosis in patients with dementia. Am J Psychiatry. 2016;173(5):543-546. doi:10.1176/appi.ajp.2015.173501 27133416

[14] Centers for Medicare & Medicaid Services. National partnership to improve dementia care in nursing homes: antipsychotic medication use data report. April 2019. Accessed December 10, 2019. https://www.cms.gov/Medicare/Provider-Enrollment-and-Certification/SurveyCertificationGenInfo/Downloads/Antipsychotic-Medication-Use-Data-Report.pdf

[15] Centers for Medicare & Medicaid Services. State operations manual: appendix PP—guidance to surveyors for long term care facilities. 2023. Accessed March 11, 2024. https://www.cms.gov/Regulations-and-Guidance/Guidance/Manuals/downloads/som107ap_pp_guidelines_ltcf.pdf

[16] Avorn J, Soumerai SB, Everitt DE, . A randomized trial of a program to reduce the use of psychoactive drugs in nursing homes. N Engl J Med. 1992;327(3):168-173. doi:10.1056/NEJM199207163270306 1608408

[17] Thompson A, Sullivan SA, Barley M, . The DEBIT trial: an intervention to reduce antipsychotic polypharmacy prescribing in adult psychiatry wards—a cluster randomized controlled trial. Psychol Med. 2008;38(5):705-715. doi:10.1017/S003329170700147X 17825122

[18] Rubino A, Sanon M, Ganz ML, . Association of the US Food and Drug Administration antipsychotic drug boxed warning with medication use and health outcomes in elderly patients with dementia. JAMA Netw Open. 2020;3(4):e203630. doi:10.1001/jamanetworkopen.2020.3630 32343351 PMC7189225

[19] Brodaty H, Aerts L, Harrison F, . Antipsychotic deprescription for older adults in long-term care: the HALT Study. J Am Med Dir Assoc. 2018;19(7):592-600.e7. doi:10.1016/j.jamda.2018.05.002 29941156

[20] Chappell V, Kirkham J, Seitz DP. Association between long-term care facility staffing levels and antipsychotic use in US long-term care facilities. J Am Med Dir Assoc. 2022;23(11):1787-1792.e1. doi:10.1016/j.jamda.2022.06.029 35926573

[21] Walsh KA, Dennehy R, Sinnott C, . Influences on decision-making regarding antipsychotic prescribing in nursing home residents with dementia: a systematic review and synthesis of qualitative evidence. J Am Med Dir Assoc. 2017;18(10):897.e1-897.e12. doi:10.1016/j.jamda.2017.06.032 28807433

[22] Centers for Medicare & Medicaid Services. Medicare Part D spending by drug. 2023. Accessed June 2, 2023. https://data.cms.gov/jsonapi/node/dataset/63b6ee35-4593-4525-a9cc-4d39cd1e9132

[23] Gerlach LB, Kales HC, Kim HM, . Trends in antipsychotic and mood stabilizer prescribing in long-term care in the U.S.: 2011-2014. J Am Med Dir Assoc. 2020;21(11):1629-1635.e8. doi:10.1016/j.jamda.2020.05.039 32693995 PMC7641905

[24] Sacarny A, Barnett ML, Le J, Tetkoski F, Yokum D, Agrawal S. Effect of peer comparison letters for high-volume primary care prescribers of quetiapine in older and disabled adults: a randomized clinical trial. JAMA Psychiatry. 2018;75(10):1003-1011. doi:10.1001/jamapsychiatry.2018.1867 30073273 PMC6233799

[25] Sacarny A, Harnisch M, Barnett ML. The effect of de-prescribing antipsychotics on health and quality of life for people with dementia. January 12, 2022. Accessed May 23, 2023. https://osf.io/eh47k/

[26] Pham HH, Schrag D, O’Malley AS, Wu B, Bach PB. Care patterns in Medicare and their implications for pay for performance. N Engl J Med. 2007;356(11):1130-1139. doi:10.1056/NEJMsa063979 17360991

[27] McWilliams JM, Chernew ME, Landon BE, Schwartz AL. Performance differences in year 1 of pioneer accountable care organizations. N Engl J Med. 2015;372(20):1927-1936. doi:10.1056/NEJMsa1414929 25875195 PMC4475634

[28] Thomas KS, Dosa D, Wysocki A, Mor V. The Minimum Data Set 3.0 cognitive function scale. Med Care. 2017;55(9):e68-e72. doi:10.1097/MLR.0000000000000334 25763665 PMC4567556

[29] McCreedy E, Ogarek JA, Thomas KS, Mor V. The Minimum Data Set agitated and reactive behavior scale: measuring behaviors in nursing home residents with dementia. J Am Med Dir Assoc. 2019;20(12):1548-1552. doi:10.1016/j.jamda.2019.08.030 31678075 PMC7008595

[30] Bélanger E, Thomas KS, Jones RN, Epstein-Lubow G, Mor V. Measurement validity of the Patient-Health Questionnaire-9 in US nursing home residents. Int J Geriatr Psychiatry. 2019;34(5):700-708. doi:10.1002/gps.5074 30729570 PMC6459696

[31] Levis B, Benedetti A, Thombs BD; DEPRESsion Screening Data (DEPRESSD) Collaboration. Accuracy of Patient Health Questionnaire-9 (PHQ-9) for screening to detect major depression: individual participant data meta-analysis. BMJ. 2019;365:l1476. doi:10.1136/bmj.l1476 30967483 PMC6454318

[32] Jenny Wei YJ, Chen C, Fillingim RB, . Uncontrolled pain and risk for depression and behavioral symptoms in residents with dementia. J Am Med Dir Assoc. 2021;22(10):2079-2086.e5. doi:10.1016/j.jamda.2021.05.010 34089652 PMC8478709

[33] Kahan BC, Jairath V, Doré CJ, Morris TP. The risks and rewards of covariate adjustment in randomized trials: an assessment of 12 outcomes from 8 studies. Trials. 2014;15(1):139. doi:10.1186/1745-6215-15-139 24755011 PMC4022337

[34] Abadie A, Athey S, Imbens GW, Wooldridge JM. When should you adjust standard errors for clustering? Q J Econ. 2022;138(1):1-35. doi:10.1093/qje/qjac038

[35] Aigbogun MS, Stellhorn R, Hartry A, Baker RA, Fillit H. Treatment patterns and burden of behavioral disturbances in patients with dementia in the United States: a claims database analysis. BMC Neurol. 2019;19(1):33. doi:10.1186/s12883-019-1260-3 30819136 PMC6396493

[36] Hallsworth M, Chadborn T, Sallis A, . Provision of social norm feedback to high prescribers of antibiotics in general practice: a pragmatic national randomised controlled trial. Lancet. 2016;387(10029):1743-1752. doi:10.1016/S0140-6736(16)00215-4 26898856 PMC4842844

[37] Sacarny A, Yokum D, Finkelstein A, Agrawal S. Medicare letters to curb overprescribing of controlled substances had no detectable effect on providers. Health Aff (Millwood). 2016;35(3):471-479. doi:10.1377/hlthaff.2015.1025 26953302

[38] Gaugler JE, Yu F, Krichbaum K, Wyman JF. Predictors of nursing home admission for persons with dementia. Med Care. 2009;47(2):191-198. doi:10.1097/MLR.0b013e31818457ce 19169120

[39] Mor V, Zinn J, Angelelli J, Teno JM, Miller SC. Driven to tiers: socioeconomic and racial disparities in the quality of nursing home care. Milbank Q. 2004;82(2):227-256. doi:10.1111/j.0887-378X.2004.00309.x 15225329 PMC2690171

[40] Rivera-Hernandez M, Kumar A, Roy I, Fashaw-Walters S, Baldwin JA. Quality of care and outcomes among a diverse group of long-term care residents with Alzheimer’s disease and related dementias. J Aging Health. 2022;34(2):283-296. doi:10.1177/08982643211043319 34634973 PMC8957613

[41] Smith DB, Feng Z, Fennell ML, Zinn JS, Mor V. Separate and unequal: racial segregation and disparities in quality across U.S. nursing homes. Health Aff (Millwood). 2007;26(5):1448-1458. doi:10.1377/hlthaff.26.5.1448 17848457

[42] Wei YJJ, Solberg L, Chen C, . Agreement of Minimum Data Set 3.0 depression and behavioral symptoms with clinical diagnosis in a nursing home. Aging Ment Health. 2021;25(10):1897-1902. doi:10.1080/13607863.2020.1758921 32447973 PMC7686050

[43] Noyes K, Liu H, Lyness JM, Friedman B. Medicare beneficiaries with depression: comparing diagnoses in claims data with the results of screening. Psychiatr Serv. 2011;62(10):1159-1166. doi:10.1176/ps.62.10.pss6210_1159 21969642

