# Supplementary material for: Physician Antipsychotic Overprescribing Letters and Cognitive, Behavioral, and Physical Health Outcomes Among People With Dementia: A Secondary Analysis of a Randomized Clinical Trial
Source: JAMA Netw Open. 2024 Apr 25;7(4):e247604. doi: 10.1001/jamanetworkopen.2024.7604 (PMC11046341; doi:10.1001/jamanetworkopen.2024.7604)
Supplement: Supplement 1. — Trial Protocol and Statistical Analysis Plan [file jamanetwopen-e247604-s001.pdf]

Effect of Physician Antipsychotic Overprescribing Letters on Cognitive, Behavioral, and Physical Health Outcomes Among People with Dementia

A Secondary Analysis of a Randomized Clinical Trial

Supplement 1: Study Methodology and Analysis Plan Supplement

Michelle Harnisch, MS; Michael L. Barnett, MD, MS; Stephen Coussens, PhD; Kali S. Thomas, PhD; Mark Olfson, MD, MPH; Kiros Berhane, PhD; Adam Sacarny, PhD

<u>Table of Contents</u>

Sample Construction **3**
Outcomes **4**
Prescribing 4
Nursing home outcomes 5
Claims-based and additional outcomes 6
Statistical Approach **7**
Subgroup Analyses **8**
References **10**

Table S1 – List of Drugs in Each Class 11
Figure S1 – Reproduction of Treatment Group Letter 12

The description of our study design closely follows and is in parts identical to the original analysis plan of this study archived online on August 2, 2022.[1] For a small number of secondary study outcomes, we deviated from the original analysis plan. All modifications to the original analysis plan are noted in the sections below.

This document also reproduces the letter that was sent to study participants in the treatment group (Figure S1 in this Supplement).

Sample Construction

We assembled repeated cross-sections of patients of study primary care physicians (PCPs), dividing them into treated patients (patients of treated study PCPs) and control patients (patients of control study PCPs). To do so, we measured patients' outcomes during 90-day periods relative to study initiation on April 20, 2015. Each measurement period yields one cross section of patients. To determine that someone is a patient of a study PCP, we used an attribution algorithm based on their claims in the 90 days prior to that period for nursing home patients (180 days for patients living in the community). Thus, each cohort of nursing home patients has a 90-day attribution period immediately prior to its 90-day measurement period and each cohort of community-dwelling patients has a 180-day attribution period immediately prior to its 90-day measurement period.

To enter the analysis sample, the patient must meet the following criteria during the measurement period:

- Enrolled in Medicare Parts A, B, and D until the end of the period or until death, whichever comes first.
- Enrolled in fee-for-service Medicare until the end of the period or death, whichever comes first.
- For nursing home patients: At least one Minimum Data Set (MDS) OBRA-required assessment during the period.[2]

The patient must also meet the following criteria during the attribution period:

- Enrolled in Medicare Parts A, B, and D during all months of the attribution period.
- Enrolled in fee-for-service Medicare during all months of the attribution period.
- For nursing home patients: Residing in a nursing home or skilled nursing facility on the last day of the period, but not on a Medicare Part A covered skilled nursing stay on this day.
- For community-dwelling patients: Not residing in a nursing home or skilled nursing facility on the last day of the period.

[1] The plan is available at https://osf.io/v2axg/
[2] We include all OBRA-required assessments conducted at admission, on a quarterly or annual basis, and upon a significant change in status. In a clarification of the analysis plan, we omit OBRA-required assessments at discharge because the vast majority of these assessments are missing key data like the depression screen and cognitive function screen.

- Diagnosed with Alzheimer's disease and/or related dementias (ADRD), defined as satisfying either of the following conditions 1) the most recent ADRD flag in the Chronic Conditions segment of the Master Beneficiary Summary File prior to the beginning of the outcome measurement period is active or 2) the date of first ever occurrence of ADRD is during the attribution period.
- Survived the period (i.e., no date of death on file, or date of death is after the period).

To attribute each patient to a single study PCP, we consider evaluation and management (E&M) services during the attribution period. For nursing home patients, the E&M place of service must be in a nursing home or skilled nursing facility. We omit hospital and emergency room E&M procedure codes.

For each patient-practitioner with at least one service, we count the total E&M allowed charges and total E&M service lines.[3] The algorithm uses the following approach to select each patient's attributed practitioner:

1. Select the practitioner(s) with the most E&M allowed charges. If there is a tie, move to the next step.
2. Among the remaining practitioners, select the one(s) with the most E&M service lines. If there is a tie, move to the next step.
3. Among the remaining practitioners, use a random tiebreak to pick one.

Only patients who were attributed to a study PCP are included in the analysis. In the rare event that a patient is attributed to multiple study PCPs across post-intervention periods, we exclude the patient from the regression analysis. We further exclude patients from the analysis of baseline characteristics if they were attributed to multiple study PCPs across pre-intervention periods.

Most patients in our sample (99.3 percent across pre- and post-intervention periods) were attributed to their practitioner based on E&M allowed charges in step one. On average, the attributed PCP was responsible for 77.4 percent of all E&M allowed charges in the nursing home sample and for 65.1 percent of charges in the community patient sample.

<u>Outcomes</u>

Prescribing

To construct prescribing endpoints, we used the 2015-2018 Prescription Drug Event Research Identifiable File (PDE RIF). For each period, we considered all Prescription Drug Events (PDEs) with a service date within the time period. Our primary endpoint was the days of quetiapine received, defined as the total number of days' supply of quetiapine across all of the patient's PDEs during the period. Secondary endpoints included binary receipt of any quetiapine and quetiapine milligrams received. Any receipt was defined as having at least one PDE for

[3] We define practitioners based on the rendering National Provider Identifier (NPI) in claims. Advanced practice providers (APPs) who bill Medicare under their own NPI are therefore eligible for attribution. Some APPs engage in "incident-to" billing in which services are billed under the NPI of a supervising physician. In such cases, we assign the services to the supervising physician.

quetiapine in the period. We calculated milligrams dispensed as the quantity dispensed multiplied by the strength value reported for quetiapine in the IBM Micromedex Red Book.

To study potential substitution patterns, prescribing endpoints further included the receipt of all antipsychotics (days, any, and milligrams) and receipt of other psychoactive medication drug classes, including antidepressants, benzodiazepines, nonbenzodiazepine sedative-hypnotics, and mood stabilizers (days and any). A list of drugs included in each of these classes can be found in Table S1 in this Supplement. To be able to compare milligrams dispensed across different antipsychotic drugs, for this endpoint we transformed the strength value of each drug into risperidone milligram equivalents using conversion factors specified by Leucht et al. (2016; 2020). For nursing home residents we additionally included a measure from the MDS, defined as the number of days the patient received antipsychotics during the last 7 days.

We made slight modifications to our original analysis plan. First, we evaluated days received of antipsychotics other than quetiapine. This outcome illustrates more clearly potential substitution patterns away from quetiapine than receipt of all antipsychotics. Second, we assessed the receipt of gabapentinoids, a class of psychoactive medication that has come under increasing scrutiny for its use in dementia patients in nursing homes (Candon et al. 2023). Finally, we evaluated whether patients received any other psychoactive medication in addition to analyzing receipt of each of the different psychoactive drug classes on its own.

Nursing home outcomes

We construct nursing home outcomes based on information from MDS assessments. Given our focus on long-stay patients, we only use assessments mandated by law under the Omnibus Budget Reconciliation Act (OBRA). These assessments must be conducted on a quarterly basis, upon a patient's admission, and when a significant change in status occurs. Assessments upon discharge are also required under OBRA, but we omit them because they have extremely high rates of missing data for our main outcomes. Patients' depression screen, for instance, is not available for 84 percent of discharge assessments and for 68 percent of discharge assessments, we do not observe patients' cognitive function.

Nursing home outcomes include measures of cognitive and behavioral health. We measured patients' cognitive function with the Cognitive Function Scale (CFS, Downer et al. 2017; Thomas et al. 2017). The CFS is a composite based on questions asked during the Brief Interview for Mental Status conducted during assessments. If the nursing home resident was not able to take part in the interview or if the interview was attempted but not completed, the score is calculated using the Cognitive Performance Scale which is based on staff observation.

We further used information from the Patient Health Questionnaire (PHQ-9) to evaluate changes in depressive symptoms (Bélanger et al. 2019). If no interview could be conducted during the assessment or the interview was started but not completed, we use the staff assessment of resident mood from the PHQ-9-OV instead. We defined positive screening for depression as having a PHQ-9 score ≥ 10 (Levis, Benedetti, and Thombs 2019; Jenny Wei et al. 2021) and also assessed the linear score itself. We also investigated the presence of any depressive symptoms,

i.e. PHQ-9 score > 0, and mild depressive symptoms, i.e., PHQ score from 5-9 (Kroenke, Spitzer, and Williams 2001).

To measure behavioral symptoms, we further constructed Agitated and Reactive Behavior Scale (ARBS) scores as well as an indicator for exhibiting any agitated or reactive behavior (ARBS>0) (McCreedy et al. 2019).

To measure delirium, we used the Confusion Assessment Method scoring methodology and the Signs and Symptoms of Delirium MDS questions. For assessments with valid responses to these questions, we defined a binary indicator for delirium. These analyses were not pre-specified.

Additionally, we analyzed whether patients received any recreational or psychological therapy and whether any restraints were used (including bed rails). We measured patients' BMI from their reported height and weight. Finally, we evaluated weight loss using a field in the assessments which identifies patients who lost 5% or more weight during the last month or 10% or more weight during the last 6 months.

For each 90-day period, we used all assessments with target dates within the period. If a nursing home resident had more than one assessment during the period, we aggregated outcomes across different assessments by using the greatest severity of impairment, symptoms, or behavior across the assessments. For receipt of therapy and use of restraints we measured whether any such services or care occurred across the assessments. For patients' BMI and the number of days the patient received antipsychotics during the last 7 days, we calculated the mean across assessments.

Claims-based and additional outcomes

To identify if a patient had a diagnosis of hyperlipidemia, hypertension, hyperglycemia, diabetes, or depression, we searched the Carrier, Outpatient, and MedPAR (i.e. inpatient and skilled nursing) claims data within each 90-day period. We used diagnosis codes associated with each of the above conditions from the Chronic Conditions Data Warehouse (CCW) algorithms.[4] The CCW provides algorithms for identifying 27 chronic conditions based on ICD-9 and ICD-10 codes. Diagnoses codes for hyperglycemia are not available from the CCW; we use ICD-9 codes 790.2X and ICD-10 codes R73.0X and R73.9 instead.

Other claims-based outcomes are any inpatient stay during the period, and any emergency department stays during the period, as well as any hospitalization for a mental health principal diagnosis or for a stroke or transient ischemic attack. We identify mental health diagnoses using all diagnoses codes associated with CCS codes 650 to 652, 655 to 659, 662, 663, and 670 following Heslin et al. (2015). Stroke or transient ischemic attack are classified using the inclusion and exclusion diagnosis codes specified in the CCW algorithm for this condition. We also included death as an outcome using date of death reported in the MBSF.

Lastly, we considered indicators for having any encounter with certain groups of clinicians during the period: the attributed study PCP, primary care practitioners, geriatricians,

[4] See https://www2.ccwdata.org/web/guest/condition-categories-chronic

psychiatrists, or psychologists. We classified practitioners based on their taxonomy information from the National Plan and Provider Enumeration System (NPPES).

Primary care practitioners were identified by a primary taxonomy starting with 207Q (family medicine), 207R (internal medicine) or 208D (general practice). For psychologists, we used primary taxonomies starting with 103T. If no primary taxonomy was available, we used the first taxonomy in the clinician's NPPES record. Geriatricians and psychiatrists were identified using all available taxonomies. Geriatrician taxonomies were defined as those starting with 207QG0300X or 207RG0300X. Psychiatrist taxonomies were those starting with 2084P08.

Clinician encounters were based on claims in the Carrier file. We excluded encounters with a place of service of inpatient hospital, emergency room, inpatient psychiatric facility, or inpatient rehabilitation facility. Since we already report results for encounters with psychologists, we deviate from the analysis plan and do not separately measure the receipt of psychotherapy in claims.

Statistical Approach

The randomization of the intervention allows us to use a simple research design to estimate causal effects (intention to treat) of the warning letters on patients' outcomes. We estimated linear regression models on the pooled data of the form:

$$y_j = \alpha_p + \beta * TREAT_{i(j)} + X_{i(j)}\Gamma + Z_j\Delta + \varepsilon_j$$

where j indexes the patients, p indexes the outcome measurement periods, and i indexes the study PCPs. y_j is the outcome of interest, $TREAT_{i(j)}$ indicates whether a patient's attributed study PCP was in the treatment group of the original study, $X_{i(j)}$ and Z_j are vectors of adjusters at the attributed PCP and patient level, respectively, and ε_j is a disturbance term. We clustered all standard errors at the level of study PCPs, which matches the level of randomization.

In Z_j, we include all outcomes displayed in Table 2 and eTable 4 measured for the patient in the last period before the intervention began. We note that in some cases, these measures may not be available for the patient. For instance, they may not have had FFS coverage, or for nursing home assessment measures, they may not have entered a nursing home yet. In these cases, we set the measure to zero. In turn, we include in Z_j indicators for whether each measure was recoded to zero because it was not available.

We further include in Z_j an indicator for the patient having Part D coverage during the last pre-intervention period and an indicator for having FFS Parts A and B coverage at that time. For the nursing home sample we also include an indicator for living in a nursing home at that time. Finally, Z_j includes patient sociodemographic factors: age, race, Hispanic/Latino/Latinx ethnicity, and sex as reported in the Medicare Master Beneficiary Sum Files. Age is included using indicators for 5-year bins grouping everyone older than 100 years and younger than 55 years together. All other sociodemographic factors are included as indicators. For race and

Hispanic/Latino/Latinx ethnicity, we use the categories reported in the Research Triangle Institute (RTI) race code.

We use X_i to adjust for baseline PCP-level outcomes. Specifically, it contains the average outcomes of patients attributed to study PCP i across all pre-intervention periods. We only consider the PCP's nursing home patients in the nursing home sample and their community-dwelling patients in the community sample. The adjusters consist of baseline averages of all outcomes in Table 2 and eTable 4. For each adjuster, if the average could not be calculated because it was not observed for any patients, we set the value to zero. We additionally include indicators for whether each average had to be recoded to zero. Finally, we include an indicator for whether the PCP had no patients during this period.

For additional outcomes displayed in eTables 2 and 3, we further included baseline values of the outcome itself at the prescriber and patient level, as well as indicators for whether these baseline measures had to be recoded to zero because they were missing.

Subgroup Analyses

We also tested for effects of the intervention on quetiapine receipt and health outcomes among a number of patient subgroups (Figure 2, eFigures 2-4). We ran separate subgroup analyses for nursing home and community patients. These analyses were exploratory and were not pre-specified.

The subgroups were as follows:

- Age in bins: <65, 65-74, 75-84, 85-94, 95+
- Race-ethnicity: white, Black, Hispanic/Latino, or other race
- Sex: male or female
- Medicare-Medicaid dual eligibility status: non-dual or dual

For the nursing home sample, we further considered the following subgroups based on nursing home indicators:

- Antipsychotic prescribing to long-stay residents: at/below median or above median
- Safety inspection star rating: at/below median or above median
- Staffing star rating: at/below median or above median

We took these indicators from the April 2015 archive of Medicare Care Compare nursing data so that they did not reflect any potential impacts of the intervention. To calculate a nursing home's antipsychotic prescribing rate, we took the average of quality measures for 2014Q2, 2014Q3, and 2014Q4, the most recent measures reported in that archive. We used the safety inspection and staffing star ratings reported in that archive.

To run the analysis for each subgroup categorization, we fully interacted the main regression with the subgroup category indicators. Then, we reported results for the subgroup-specific treatment effect (i.e. the main treatment effect plus the treatment-subgroup interaction effect). This approach is essentially equivalent to running the main regression subgroup-by-subgroup,

but it more easily facilitates Wald tests of the null hypothesis that effects were constant across the subgroups.

References

Bélanger, Emmanuelle, Kali S. Thomas, Richard N. Jones, Gary Epstein-Lubow, and Vincent Mor. 2019. “Measurement Validity of the Patient-Health Questionnaire-9 in US Nursing Home Residents.” *International Journal of Geriatric Psychiatry* 34 (5): 700–708. https://doi.org/10.1002/gps.5074.

Candon, Molly, Julie Strominger, Lauren B. Gerlach, and Donovan T. Maust. 2023. “Antiepileptic Prescribing to Persons Living with Dementia Residing in Nursing Homes: A Tale of Two Indications.” *Journal of the American Geriatrics Society* 71 (1): 89–97. https://doi.org/10.1111/jgs.18119.

Downer, Brian, Kali S. Thomas, Vincent Mor, James S. Goodwin, and Kenneth J. Ottenbacher. 2017. “Cognitive Status of Older Adults on Admission to a Skilled Nursing Facility According to a Hospital Discharge Diagnosis of Dementia.” *Journal of the American Medical Directors Association* 18 (8): 726–28. https://doi.org/10.1016/j.jamda.2017.04.021.

Heslin, Kevin C., A. Elixhauser, and C. A. Steiner. 2015. “Hospitalizations Involving Mental and Substance Use Disorders among Adults, 2012.” Agency for Healthcare Research and Quality (US), Rockville (MD). https://europepmc.org/article/NBK/nbk310986.

Jenny Wei, Yu-Jung, Cheng Chen, Roger B. Fillingim, Steven T. DeKosky, Siegfried Schmidt, Marco Pahor, Laurence Solberg, and Almut G. Winterstein. 2021. “Uncontrolled Pain and Risk for Depression and Behavioral Symptoms in Residents With Dementia.” *Journal of the American Medical Directors Association* 22 (10): 2079-2086.e5. https://doi.org/10.1016/j.jamda.2021.05.010.

Kroenke, Kurt, Robert L. Spitzer, and Janet B. W. Williams. 2001. “The PHQ-9: Validity of a Brief Depression Severity Measure.” *Journal of General Internal Medicine* 16 (9): 606–13. https://doi.org/10.1046/j.1525-1497.2001.016009606.x.

Leucht, Stefan, Alessio Crippa, Spyridon Siafis, Maxine X. Patel, Nicola Orsini, and John M. Davis. 2020. “Dose-Response Meta-Analysis of Antipsychotic Drugs for Acute Schizophrenia.” *American Journal of Psychiatry* 177 (4): 342–53. https://doi.org/10.1176/appi.ajp.2019.19010034.

Leucht, Stefan, Myrto Samara, Stephan Heres, and John M. Davis. 2016. “Dose Equivalents for Antipsychotic Drugs: The DDD Method.” *Schizophrenia Bulletin* 42 (suppl_1): S90–94. https://doi.org/10.1093/schbul/sbv167.

Levis, Brooke, Andrea Benedetti, and Brett D Thombs. 2019. “Accuracy of Patient Health Questionnaire-9 (PHQ-9) for Screening to Detect Major Depression: Individual Participant Data Meta-Analysis.” *BMJ*, April, l1476. https://doi.org/10.1136/bmj.l1476.

McCreedy, Ellen, Jessica A. Ogarek, Kali S. Thomas, and Vincent Mor. 2019. “The Minimum Data Set Agitated and Reactive Behavior Scale: Measuring Behaviors in Nursing Home Residents With Dementia.” *Journal of the American Medical Directors Association* 20 (12): 1548–52. https://doi.org/10.1016/j.jamda.2019.08.030.

Thomas, Kali S., David Dosa, Andrea Wysocki, and Vincent Mor. 2017. “The Minimum Data Set 3.0 Cognitive Function Scale:” *Medical Care* 55 (9): e68–72. https://doi.org/10.1097/MLR.0000000000000334.

Table S1 – List of Drugs in Each Class

Class	Drugs
Antipsychotics	Aripiprazole, Asenapine, Brexpiprazole, Cariprazine, Chlorpromazine, Clozapine, Fluphenazine, Haloperidol, Iloperidone, Loxapine, Lurasidone, Molindone, Olanzapine, Paliperidone, Perphenazine, Pimavanserin, Pimozide, Quetiapine, Risperidone, Thioridazine, Thiothixene, Trifluoperazine, Ziprasidone
Antidepressants	Amitriptyline, Amoxapine, Buproprion, Citalopram, Clomipramine, Desipramine, Desvenlafaxine, Doxepin, Duloxetine, Escitalopram, Fluoxetine, Fluvoxamine, Imipramine, Isocarboxazid, Maprotiline, Milnacipran, Mirtazapine, Nefazodone, Nortriptyline, Paroxetine, Phenelzine, Protriptyline, Selegiline, Sertraline, Tranylcypromine, Trazodone, Trimipramine, Venlafaxine, Vilazodone
Benzodiazepines	Alprazolam, Chlordiazepoxide, Clobazam, Clonazepam, Clorazepate, Diazepam, Estazolam, Flurazepam, Halazepam, Lorazepam, Oxazepam, Prazepam, Quazepam, Temazepam, Triazolam
Gabapentinoids	Gabapentin, Pregabalin
Mood stabilizers	Carbamazepine, Divalproex sodium, Felbamate, Lamotrigine, Lithium, Oxcarbazepine, Topiramate, Valproate sodium, Valproic acid
Nonbenzodiazepine sedative-hypnotics	Doxepin, Eszopiclone, Ramelteon, Suvorexant, Tasimelteon, Zaleplon, Zolpidem

Figure S1 – Reproduction of Treatment Group Letter

Page 1 of 3

Department of Health & Human Services
7500 Security Boulevard, Mail Stop AR-18-50
Baltimore, Maryland 21244-1850

CMS
CENTERS FOR MEDICARE & MEDICAID SERVICES
CENTER FOR PROGRAM INTEGRITY

April 20, 2015

Pat Q. Provider MD
1234 Main St
Columbia, MD 21045
NPI: 1234567890 / Specialty: General Care Practitioner

Re: Your Seroquel prescribing is under review by the Center for Program Integrity.

Dear Dr. Provider,

The figure to the right displays your prescribing of Seroquel treatments (Seroquel, Seroquel XR, or generic quetiapine) compared to other general care practitioners in Maryland.

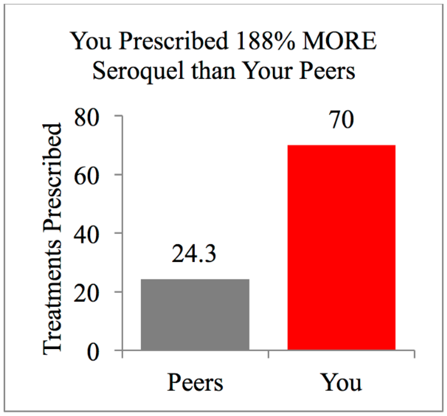

As can be seen, **you prescribed far more treatments – 188% more – than similar prescribers within your state.** In turn, you have been flagged as a markedly unusual prescriber, subject to review by the Center for Program Integrity.

We recognize that some flagged practitioners have appropriate reasons for this pattern. However, we have seen that other practitioners may drift into prescribing patterns that would be considered medically unjustified or abusive. Abusive prescribing can lead to extensive audits and even revocation of Medicare billing privileges.

We hope that you will use this information to see if your high prescribing level is consistent with the latest standards of care. To assist in your monitoring efforts, CMS will periodically send you letters with our most recent information about your Seroquel prescribing. **We may contact you at a later date to ask what steps, if any, you have taken in response to our communications.**

Read on for more information about the methodology used to analyze your prescribing behavior and to learn what actions to take next.

Sincerely,

[redacted]

Investigations and Audit Group

Figure S1 cont. – Reproduction of Treatment Group Letter

Page 2 of 3

Introduction

Prescribers and pharmacies have a frontline role in assisting the Centers for Medicare & Medicaid Services (CMS) to effectively manage Medicare resources and monitor prescribing practices. CMS and its partners acknowledge the daily challenges prescribers and pharmacies face in serving Medicare beneficiaries and the complexity of billing for prescription drugs.

The Office of Inspector General (OIG) released a study in June 2013 showing over 1 million individual prescribers ordered drugs paid by Medicare Part D in 2009. Prescribing patterns varied widely by type of prescriber. Over 700 general-care physicians had questionable prescribing patterns.[1] Although some of this prescribing may be appropriate, the OIG's study expressed the need to further scrutinize such questionable patterns.

Using a similar methodology to that used in the OIG study, CMS analyzed Seroquel, Seroquel XR, and generic quetiapine prescribing data for calendar year 2013 and 2014.[2] Based on this analysis, CMS has determined that the number and quantity of your treatments exceeded the established threshold (see box on right). The intent of this letter is to inform you of the extent of your potential outlier status, which reflects the Seroquel treatments attributed to your prescribing practice compared to your peers within your prescriber type (general care practitioner) and state (Maryland).

More Information

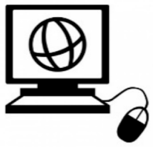

To learn more about Seroquel prescribing and to find details on our methodology, visit (this link is case sensitive): http://go.cms.gov/CPImethodologyhr2

We hope you find this information helpful and that it will provide insights into your current and future prescribing practices. We also hope that you will use the information provided to see if your high prescribing level for Seroquel is appropriate for your patient population.

Your Results

Table 1: Values Used to Determine Your Status as a Potential Outlier

Categories	Total Treatments	30-Day Equivalent
Your Values in 2014	70	79
Average of General Care Practitioners in Maryland	24.3	26.7

This table reviews the data that led to your classification as a potential outlier.

[1] OIG, Prescribers with Questionable Patterns in Medicare Part D, OEI-02-09-00603, June 2013

[2] The prescribing data consists of Prescription Drug Event (PDE) records. Each PDE is a summary record submitted by a drug plan sponsor whenever a beneficiary fills a prescription under Medicare Part D. The PDE data are not the same as individual drug claim transactions, but are summary extracts using CMS-defined standard fields. The PDE record contains prescription drug cost and payment data that enables CMS to make payments to plans and otherwise administer the Part D benefit. Further information can be found by accessing the following link: http://www.cms.gov/Medicare/Prescription-Drug-Coverage/PrescriptionDrugCovGenIn/PartDData.html

Figure S1 cont. – Reproduction of Treatment Group Letter

Page 3 of 3

Total Treatments displays the total number of treatments for Seroquel filled by your patients. A Seroquel treatment is defined as a patient visit to a pharmacy in which a Seroquel, Seroquel XR, or generic quetiapine prescription is filled. This data is shown in the graph on the first page of this letter. **30-Day Equivalent** reports the number of treatments adjusted by the days' supply of the drug dispensed.

Action

After reviewing this communication, you may be able to identify areas where your prescribing patterns could be modified, and we encourage you to share the trends that were identified with other clinicians. We hope you find this information helpful and that it will provide insight into your current and future prescribing practices.

If you would like to discuss this project or your data, or provide feedback on this analysis, please contact the NBI MEDIC at 1-877-7SafeRx (1-877-772-3379) or CMS at CPIMedicarePartD_Data@cms.hhs.gov. If you believe your prescriptions are being forged, please contact the NBI MEDIC at 1-877-7SafeRx (1-877-772-3379).

If you would like more resources for detecting possible drug-seeking behavior on the part of your patients, please review the MLN Matters article on Prescription Drug Monitoring Programs (PDMPs) at https://www.cms.gov/Outreach-and-Education/Medicare-Learning-Network-MLN/MLNMattersArticles/Downloads/SE1250.pdf on the CMS website.

Thank you for your diligence and partnership with CMS in detecting, deterring and preventing fraud, waste and abuse in the Medicare Part C and Part D programs.

Protocol of Original Trial

Peer Comparison Letters for High Volume Primary Care Prescribers of Quetiapine in Older and Disabled Adults: A Randomized Clinical Trial

Supplement 1: Study Methodology and Extended Results

Adam Sacarny, PhD; Michael L. Barnett, MD, MS; Jackson Le, PharmD; Frank Tetkoski, RPh; David Yokum, PhD; Shantanu Agrawal, MD

Table of Contents

STUDY METHODOLOGY AND INTERVENTION 3
Identification of Prescribers and Randomization 3
Intervention 4
Control Group Intervention 5
Treatment Group Intervention 5

ANALYSIS PLAN AND EXTENDED RESULTS 6
Statistical Approach 6
Regression Control Variables 7
Outcome Measurement Period (Horizon) 8
Results 9
Prescriber-Level Outcomes 9
Total Quetiapine Prescribing 9
New and Continuing Prescribing 9
Prescribing to Low-Value and Guideline-Concordant Patients 10
Characteristics of the Average Patient 10
Heterogeneous Effects on Prescribers 11
Quantile Effects 11
Prescribing of Other Psychiatric Drugs 12
Patient Level Outcomes 13
Patient Receipt of Quetiapine 13
Quantile Effects 14
Heterogeneous Effects on Patients 15
Effects for Low-Value and Guideline-Concordant Patients by Source 15
Receipt of Other Psychiatric Drugs 16
Patient Mortality and Health Care Utilization 17
Sensitivity and Robustness 18
References 20
Tables 21
Pre-Intervention Data 21
Effects on Prescriber-Level Outcomes 22
Effects on Patient-Level Outcomes 27
Sensitivity and Robustness Tests 33
Attachments 36

STUDY METHODOLOGY AND INTERVENTION

In this section we describe the intervention for prescribers of quetiapine in full detail. We review the methodology by which the prescribers were identified and the mailings that prescribers in each group were sent.

Identification of Prescribers and Randomization

We conducted an analysis to identify outlier prescribers of quetiapine in the Medicare Part D event file for each of two time periods, calendar year 2013 (called universe 1) and calendar year 2014 (called universe 2). We define quetiapine prescriptions as prescriptions for branded Seroquel, Seroquel XR, or generic quetiapine.

First, for each universe, all general-care prescribers (which includes General Practitioners, Family Practitioners, and Internal Medicine practitioners; practitioners with a secondary or tertiary specialty of Psychiatry were excluded) with at least 10 quetiapine prescription drug events (PDEs, or records in the Part D events file that are generated whenever patients fill prescriptions) were identified. Prescriptions filled through long-term care pharmacies and filled to beneficiaries residing in institutional settings were then removed. This category of fills was included in all data collected to analyze the intervention, however.

Prescribers were then grouped by state (e.g. a prescriber's peer group was other prescribers in his/her state) and two outlier thresholds were calculated for each group. In order to be considered an outlier, the prescriber had to pass both thresholds.

The first threshold was with respect to quetiapine prescription drug treatments, or PDT. A quetiapine PDT rolls up all the PDE for a given prescriber-beneficiary-day into one treatment; multiple PDE delivered to a beneficiary from one prescriber on the same day only count as one PDT. The threshold was set equal to the 75th percentile for prescribers within the state plus 0.25 times the interquartile range (called the Tukey method; see Tukey, 1977).

The second threshold was with respect to quetiapine 30-day equivalent PDTs – the total "days supply" of each PDT (which equals the maximum "days supply" of the PDEs included in the PDT), divided by 30. The threshold for 30-day equivalents was set by the same Tukey method.

When this analysis was conducted using PDT and 30-day equivalent PDT from universe 1, 7,349 outlier prescribers were identified. When it was conducted using records from universe 2, 7,614 outlier prescribers were identified. 5,056 prescribers were outliers in both years; one prescriber had died by March 1, 2015 and was removed from the analysis. The remaining 5,055 prescribers became the study sample.

On March 13, 2015 we randomly allocated each of the 5,055 prescribers to a treatment or control group. Randomization was performed in Stata with a pre-specified re-randomization procedure to reject samples with large imbalances in covariates between treatment and control groups. We did not stratify the sample when randomizing.

The re-randomization procedure was as follows:

1. Random values are generated. Prescribers with values below the median are assigned to control. All others assigned to treatment.
2. We test covariate balance for a set of covariates using MANOVA, equivalent to the Mahalanobis distance (recommended in Lock Morgan and Rubin 2012). This is very similar to regressing each covariate on treatment indicator, then jointly testing all the coefficients on the treatment indicators. The covariates are:
    a. PDT Count, 2014
    b. PDT Count, 2013
    c. 30-Day PDT Count, 2014
    d. 30-Day PDT Count, 2013
    e. $ Paid for quetiapine, 2014
    f. $ Paid for quetiapine, 2013
    g. Indicator for Census Northeast (CT, ME, MA, NH, RI, VT, NJ, NY, PA)
    h. Indicator for Census Midwest (IL, IN, MI, OH, WI, IA, KS, MN, MO, NE, ND, SD)
    i. Indicator for Census West (AZ, CO, ID, MT, NV, NM, UT, WY, AK, CA, HI, OR, WA)
       Note: reference category is Census South. We included Puerto Rico in this category.
    j. Indicator for found in Medicare Part D program integrity contractor's tracking database
    k. Indicator for found in CMS Fraud Investigation Database (FID)
    l. Indicator for found in CMS Compromised Numbers Checklist (CNC)
    m. Indicator for enrolled in Medicare according to Provider Enrollment, Chain and Ownership System (PECOS) database
    n. Indicator for found in Schedule II outlier prescriber study (see Sacarny et. al., 2016)
3. If the p-value of the F test is < 0.4, return to step 2. Otherwise accept the randomization.

The first run of randomization passed the balance test so no re-randomization was conducted. 2,527 prescribers were allocated to the treatment group and 2,528 were allocated to the control group. Summary statistics about the prescribers derived from the pre-intervention data we used are shown in Table P1.

Intervention

The intervention involved one placebo letter for the control group and an initial letter and 2 followup letters for the treatment group. The role of the placebo letter was to observe letters returned to sender in the full study sample so that CMS could verify the accuracy of addresses for the prescribers. An additional notice was also sent to the control group. Prescriber addresses were drawn from the CMS PECOS and National Plan and Provider Enumeration System (NPPES) files. With permission of the institutional review boards overseeing the study, the intervention messages did not state that they were sent as part of a study (and participants were not otherwise notified of the study) to avoid contaminating the research design.

Control Group Intervention

On April 20, 2015, the control group was sent a letter describing a new Medicare regulation that would require health care providers to enroll in Medicare in order to write prescriptions in Medicare Part D (see Attachment 1). The letter also included a “Medicare Learning Network” attachment (MLN Matters Number SE1434) describing the regulation. This mailing did not mention the prescriber’s outlier quetiapine prescribing.

CMS was in the process of delaying the new regulation, and also found that some prescribers who received the letter had questions about their enrollment status. To clarify these points, the placebo group was sent a mailing during the week of June 8, 2015 (dated June 10, see Attachment 2). The mailing did not mention the prescriber’s outlier quetiapine prescribing.

Treatment Group Intervention

On April 20, 2015, the treatment group was sent a letter indicating that they were under review by CMS for their quetiapine prescribing. The letter is included in Attachment 3.

CMS then collected letters that were returned to sender. By July 1, 2015, 324 letters had been returned to CMS. 159 of these prescribers were in the treatment group. Their addresses were resolved and they were re-sent the initial letter (with refreshed data in the letter and a new date stamp) on July 9, 2015. The control group prescribers with returned correspondence were not re-sent the placebo letter.

In letters dated August 31, the treatment group prescribers were sent a followup letter updating them on their prescribing of quetiapine in 2015 using prescribing records as of June 2015 (Attachment 4). Two of the treatment group prescribers had died by this time and they were not contacted. Prescribers with 2015 quetiapine volumes below their peers or with no quetiapine prescribing in 2015 were sent a similar letter with a modified message that acknowledged this fact. Prescribers who had changed their taxonomy code received a letter with an additional paragraph acknowledging the change.

A second followup was sent to treatment prescribers in October. This followup was an updated version of the August letter, dated October 20, 2015. The letter was identical to the first followup, with the only changes being to the dates listed in the letter and that the data in it reflected prescribing through September 2015.

ANALYSIS PLAN AND EXTENDED RESULTS

In this section, we present the full results of the study following the pre-specified analysis plan that was archived on March 5, 2016, prior to unblinding to post-intervention data.[1] The contents of this section are based on, and in some cases incorporate, the text of the original analysis plan. We note all ex-post clarifications and modifications to the analysis plan.

The goal of this study is to understand the effects of the letters on both prescribers and patients. The primary outcome of the study is the effect of the letters on the prescribing of quetiapine over the 9 months following the initial sending of the letters. Prescribing is defined as the total days supply of quetiapine attributed to the prescriber (defined as Seroquel, Seroquel XR, and generic quetiapine). For the primary outcome, we define the outcome as days supply of prescription drug treatments (PDT): one treatment refers to one or more prescription fills attributed to one prescriber and one patient on a given day. Multiple quetiapine fills from the prescriber to the patient on that day do not count as additional treatments. The days supply adjustment counts the treatment with the greatest "days supply" listed on the included fills – a treatment with a 30 day supply counts as 30, while a treatment with a 15 day supply counts as 15.[2]

We consider additional outcomes as well. Through these additional analyses, we explore a broad set of the effects of the letters on prescribing and prescription drug receipt. Additional analysis for prescribers includes explorations of effects on new vs. old prescriptions, effects on the types of patients receiving prescriptions, effect heterogeneity, quantile treatment effects, and substitution toward other substances. We also conduct analyses looking at a cohort of patients who were treated by the prescribers in the year prior to the sending of the letters. As described in the text, we assign these patients to treatment and control groups based on whether their attributed prescriber was a treatment or control prescriber and study the receipt of quetiapine by patients, heterogeneity in treatment effects, and substitution toward other substances. We also study impacts on health care utilization.

We observe the behavior of prescribers and outcomes of patients through our access to the CMS Integrated Data Repository (IDR), the database used by Medicare and its program integrity contractors to detect and stop fraud, waste, and abuse. The IDR includes beneficiary enrollment information, risk-adjustment information, Medicare Part A and B claims, Medicare Part C encounter data, and Medicare Part D prescription drug events. This study uses enrollment information, risk-adjustment information, and Part D event data.

Statistical Approach

The physician level regressions are of the form:

---

[1] The original analysis plan can be accessed from the study's AEA RCT registry page, https://www.socialscienceregistry.org/trials/729

[2] The original analysis plan defined the primary outcome as 30 day equivalent PDT rather than days supply of PDT; this approach was also used in the initial analysis to identify and randomize the prescribers. The days supply metric is the 30 day equivalent metric multiplied by 30. In the main text and this supplement, for ease of exposition, we use the days supply metric.

$$y_i = \alpha + \beta * treat_i + X_i\Gamma + e_i$$

Where i indexes physicians, y_i is the outcome (e.g. number of prescriptions), $treat_i$ is an indicator for physician i being in the treatment group, and X_i is the set of physician controls. β, the effect of the treatment on the outcome, is the coefficient of interest. The standard errors for these regressions are heteroscedasticity-robust.

The patient level regressions are of the form:

$$y_j = \alpha + \beta * treat_{i(j)} + X_{i(j)}\Gamma + Z_j\Theta + e_j$$

Where j indexes patients, $i(j)$ is patient j's baseline physician, y_j is the outcome (e.g. prescriptions filled by the patient), $treat_{i(j)}$ is an indicator for the patient's physician being in the treatment group, X_i is the set of controls for characteristics of the patient's baseline physician, and Z_j is the set of controls for characteristics about the patient. β, the effect of the treatment on the outcome, is the coefficient of interest. Standard errors are clustered at the level of the baseline physician in all patient-level regressions to allow intra-physician correlations in the disturbance term.

Regression Control Variables

Since the treatment was assigned at random, the regressions produce valid estimates of the coefficients of interest even without controls. However, controls can raise power by reducing the variance of the regression error term. The analysis plan listed three specifications, with the baseline specification (reported in main tables) the one with the richest set of controls:

1. **No control variables**. This specification will yield the difference in means between the treatment and control arms.
2. **Lagged outcome controls**. The regression controls for the outcome measure in the time period before the letters were sent. That is, if the outcome is quetiapine treatments over 3 months, the control is quetiapine treatments in the 3 months before the letters were sent. In the patient level specifications, we include the lagged value of the physician outcome as well.
3. **Lagged outcome controls + additional controls (baseline specification).** The regression includes the controls of the second specification plus more information about the prescriber (and, when applicable, the patient).

Because our sample of claims began in 2013, lagged outcomes at durations longer than 1 year could not be constructed for all outcomes (some outcomes, like lookback-based new prescribing, require an additional prior year of data). As a result, for specifications 2 and 3 and outcome durations longer than 1 year, we use the 1 year lagged outcome.

For specification 3, we defined the additional controls before running the regressions. We used the following additional physician controls, which were collected in advance of the intervention going into the field:

- 30-Day equivalent treatments (days supply divided by 30) in 2013

- 30-Day equivalent treatments (days supply divided by 30) in 2014
- Indicator for entry in the CMS Fraud Investigation Database
- Indicator for entry in the Medicare Part D integrity contractor's tracking database
- Indicator for entry in the Compromised Number Checklist
- Indicator for enrollment in Medicare (PECOS)

For patient-level regressions, we additionally included the physician controls and the following patient controls:

- Age/Race/Sex interactions (age at intervention start in 5-year categories with the first category age<40 and the last category age>=90; race as white/nonwhite, and sex as female/not female)
- Indicator for being institutionalized in a long-term care facility in March 2015
- Indicators for a history of the following conditions using Medicare Part D risk-adjustment data based on 2013 and 2014 diagnosis codes. These codes were submitted by Medicare Advantage plans (for Medicare Advantage enrollees) or were collected in Medicare Part A and B claims (for Original Medicare enrollees). We provide the CMS RxHCC category we used in parentheses:
    - Alzheimer's (RxHCC 54)
    - Dementia (RxHCC 55)
    - Schizophrenia (RxHCC 58)
    - Bipolar Disorder (RxHCC 59)
    - Major Depression (RxHCC 60)
    - Specified Anxiety, Personality, and Behavior Disorders (RxHCC 61)
    - Depression (RxHCC 62)
    - Anxiety Disorders (RxHCC 63)
- Indicator for being a new beneficiary, defined as lacking risk-adjustment data in both 2013 and 2014

In the supplement, we present specifications 1 and 3. In the Sensitivity section, we show the sensitivity of the outcomes presented in Table 2 of the main text to the different specifications.

<u>Outcome Measurement Period (Horizon)</u>

In the analysis plan, we pre-specified that the primary outcome would be a count of quetiapine supplied over a 9 month duration – any fill in the 9 months following the mailing of the letters on April 21, 2015. In the main text and supplement, all secondary outcomes are therefore presented counting fills or encounters during the same time period (defined as 270 days starting on April 21, 2015) unless otherwise noted.

In the Sensitivity section, we show effects on quetiapine supply at a set of alternative durations, in 3 month increments, to 2 years. The analysis plan listed 15 and 18 month durations as optional analyses, and did not pre-specify any longer durations. However, at the most recent time of data extraction, data was reliable at durations as long as 2 years. In the main text, we therefore show this exploratory extension of outcome duration.

The plan also pre-specified that we calculate outcomes for the time periods before and after each letter was sent to look for suggestive evidence of incremental effects of additional letters. Because this evidence would be only suggestive and would not yield causal effects of the subsequent letters, we have not constructed nor studied outcomes that count quetiapine supplied during these alternative time periods.

Results

Tables P2 through P6 present the pre-specified outcomes for prescribers. Tables P7 through P12 present outcomes for patients. Tables P13 through P15 test the sensitivity of the outcomes to different specifications and measurement approaches.

All outcomes are based on the quetiapine days supply (of PDT) measure unless otherwise stated. The percent difference and adjusted difference in the tables are based on the regression specification with the full set of controls (specification 3) unless the table notes state otherwise. The adjusted difference is the coefficient on the treatment arm indicator in the regression. The percent difference is that coefficient (and its standard error) divided by the control group mean outcome. The raw difference presented in the tables is the coefficient on the treatment arm indicator from the regression specification without any controls (specification 1).

All outcomes are at the 9 month (270 day) duration, replicating the primary study outcome duration, except where stated in the sensitivity tables.

Prescriber-Level Outcomes

Total Quetiapine Prescribing

The main text presents the **primary outcome**: total quetiapine prescribing measured in days supply of PDT occurring over the 9 month duration (the 270 days beginning with April 21, 2015, the day after the initial mailing of the letters).

The prespecification document listed other ways of measuring of total quetiapine prescribing, which we consider secondary outcomes. The document also listed other outcome horizons, i.e. the same measures of quetiapine prescribing but counting prescriptions over alternative time ranges, as additional secondary outcomes. Estimates of effects on these secondary outcomes are presented in robustness tables P14 and P15, and are described in the Sensitivity and Robustness section.

New and Continuing Prescribing

Quetiapine prescriptions may have refills, allowing patients to continue receiving the drug even if their physician has begun limiting prescribing. Relatedly, physicians may focus their efforts on stopping new initiations of quetiapine prescribing, or they may try to reduce prescribing to their existing patients. To explore where physicians concentrated their efforts, we develop two approaches to differentiating between new and continuing prescribing. Table P2 presents these results. By both definitions, new prescribing declined more than continuing prescribing.

The lookback-based definition uses historical prescription drug records to define whether a prescription to a patient is new or continues an existing prescribing relationship. A new fill is one that occurs after at least 1 year of no fills for that patient attributed to the physician. Continuing fills are those that occur within 1 year of an earlier fill. In columns 2 and 3, we find that new prescribing declined by 27.1%, almost three times the decline in continuing prescribing of 9.9%. However, new prescribing only accounted for 7.6% of the control group prescribing volume, so most of the reduction in prescribing was due to a slowing of continuing prescribing.

The fill field definition uses the record's fill number (a field in the prescription drug event data) to differentiate between new and existing prescriptions. A fill that is not marked as a refill is considered new, while fills marked as refills are considered to be continuing. We de-emphasize this measure for two reasons. First, the fill field is known by CMS to be inaccurate at times. Second, this approach does not precisely differentiate between new and continuing prescribing: if a physician stops prescribing to an existing patient, both new and continuing fills will decline by the fill field definition, since when patients exhaust their refills, they must return to their physician to get a new prescription. Given this fact, new prescriptions are unsurprisingly a bigger fraction (40.4%) of baseline prescribing under the fill field approach. Still, looking to columns 4 and 5, this approach also finds a bigger reduction in new prescribing (15.0%) than continuing prescribing (8.8%).

Prescribing to Low-Value and Guideline-Concordant Patients

The main text presents and discusses our findings with respect to prescribing to low-value and guideline-concordant patients (see Table 2; eFigure 2A in Supplement 2 displays the effects over time). The prespecification document proposed that we would split patients into relatively questionable and appropriate candidates for quetiapine using claims-based indicators like whether the patient resides in a nursing home, has a history of dementia, and has a history of psychiatric disorders (we use the terms low-value and guideline-concordant in the text instead of questionable and appropriate, respectively). To operationalize this proposal, we define low-value patients as those with a history of Alzheimer's or dementia (RxHCCs 54 or 55 in Medicare Part D risk-adjustment data based on 2013 and 2014 encounters) without a history of schizophrenia, bipolar, or major depression (RxHCCs 58, 59, or 60 in the same risk-adjustment data). We define guideline-concordant patients as those with a history of schizophrenia, bipolar disorder, or major depression without a history of Alzheimer's or dementia. See eTable 1 in Supplement 2.

Characteristics of the Average Patient

In Table P3, we test how the letters affect the characteristics of the average patient who receives quetiapine from the prescriber. The characteristics are:

- Patient age
- Patient resides in a long-term care institution in March 2015 (the month prior to the intervention start)
- Patient has prior diagnosis of dementia
- Patient has prior diagnosis of psychiatric disorder

For each characteristic and each prescriber, we take the (days supply of PDT weighted) average of the characteristic. We use these averages as left-hand side measures in the regressions.

There were two statistically significant effects. First, we find that the fraction of patients residing in a long-term care institution rises by a statistically significant 0.5 percentage points or 5.7% (column 2). Second, the fraction with a history of major psychiatric disorders rises by a statistically significant 0.1 percentage points or 2.0% (column 4).

Heterogeneous Effects on Prescribers

We test for heterogeneous effects across physicians and present the results in Table P4.

In columns 2 and 3 we look across the dimension of having a previous fraud investigation – the letters may have a different effect depending on whether the doctor was already investigated. We split the sample of physicians into those who were previously investigated for fraud before the letters were sent out vs. those who were not investigated. We define fraud investigation as having an entry in the Fraud Investigation Database (FID), the Medicare Part D integrity contractor's internal database, or the Compromised Number Checklist (CNC). The prespecification document indicated that we would include prescribers flagged in the Fraud Prevention System (FPS), but we excluded it from the definition because it does not signify an investigation of fraud, but rather that the prescriber was flagged as potentially fraudulent.

The 607 prescribers with a previous investigation tend to have a higher mean volume than other prescribers (4,242 days supplied vs. 2,685 days supplied, in the respective control arms) and the intervention reduced their quetiapine prescribing by 14.7%, more than the 10.4% reduction observed for the other prescribers.

In columns 4 and 5 we split prescribers into two groups: those below or above median prescribing volume (measured in days supply) in the 9 months before the letters were sent. Columns 6 through 9 present an exploratory approach (i.e. not pre-specified) of dividing them into 4 groups on the basis of quartiles of previous prescribing volume. We find that prescribing declined in all groups regardless of the method of dividing the prescribers. The decline was greater for those above median than below (12.0% vs. 8.9%). The quartile breakdown reveals that the magnitude of the percent effect did not increase monotonically through more narrowly defined groups (the smallest magnitude occurred in quartile 2 and the largest occurred in quartile 3), though the absolute effect did.

Quantile Effects

In Table P5 we estimate quantile treatment effects to consider the impact of the letters on points of the *ex post* prescribing distribution. We look at effects on the 10th, 25th, 50th, 75th, and 90th quantiles.

The interpretation of coefficients in quantile regressions is affected by the inclusion of controls. The coefficient for the treatment variable gives the treatment effect conditional on the controls. In quantile regression, unlike linear regression, this effect does not generally equal the effect of the treatment on the unconditional distribution of the outcome (Powell 2016). It is the unconditional effect which is of interest, as this effect indicates how the point of the distribution

was affected by the intervention. Thus, in Table P5 we use no control variables, making all effects unconditional by definition. These estimates are unbiased due to the randomization.

Column 1 repeats the primary study outcome under the specification without controls (specification 1) for comparison with the subsequent quantile regressions. This linear regression approach yields an estimated decline in the average level of prescribing of 14.3%.

Columns 2 through 6 present the quantile effects. We estimate that the intervention reduced the 10th quantile of prescribing by 3.8%, the 25th quantile by 8.9%, the median by 10.3%, the 75th quantile by 11.6%, and the 90th quantile by 18.0%. The effects for the 25th quantile and above are statistically significant. Thus the intervention reduced prescribing at all points of the distribution (though we fail to reject no effect at the very bottom of the distribution); the reductions were biggest, in percent terms, at the top of the distribution.

Prescribing of Other Psychiatric Drugs

Telling physicians that their quetiapine prescribing is being monitored could trigger them to substitute their patients to other psychiatric drugs. Substitution activity could be considered gaming of the review notification if it moves patients to similar drugs with similar guideline indications and effects. For example, if prescribers moved patients to other atypical antipsychotics (drugs in the same class as quetiapine) or first generation antipsychotics (which are also not recommended for behavior control for the elderly, see American Geriatrics Society 2015), there may be little change in downstream health outcomes. On the other hand, physicians could substitute their patients to drugs that are better aligned to their conditions. For example, quetiapine is not labeled for patients with moderate depression, and a patient with moderate depression but lacking a severe mental illness diagnosis could be moved to an antidepressant drug.

In Table P6 we consider prescribing of other psychiatric drugs and find no effect on any of them, though the point estimates are consistent with a small reduction in prescribing of other antipsychotic drugs. To use a measurement that is standard across drug classes, this table presents all outcomes in the total number of days supplied of prescription drug events (PDE) rather than prescription drug treatments (PDT). These measurements both count the days supplied of the fills; they are the same except when multiple fills are dispensed to the same patient, from the same prescriber, on the same day. In this case, the days supply of PDE tallies the days supply of all of the fills, while the days supply of PDT only tallies the days supply of the fill that had the greatest days supply.

Column 1 considers quetiapine prescribing. Due to the use of PDE rather than PDT, the estimate matches the result in column 4 of Table P15 (which presents robustness of the primary outcome to alternative measures of prescribing). By this measure, quetiapine prescribing fell by 11.0%, rather than 11.1% as reported in the main text.

Columns 2 and 3 consider prescribing of other atypical antipsychotics and first generation antipsychotics, respectively, and find negative effects on both; these effects are statistically insignificant, though the point estimates are consistent with, if anything, an additional reduction in prescribing for these two categories. Column 4 considers benzodiazepine drugs commonly

prescribed for psychiatric indications like anxiety and estimates no significant effect. Columns 5 and 6 find no effect on prescribing of drugs commonly used as sleep aids looking at both the benzodiazepine and non-benzodiazepine classes, respectively. Column 7 estimates no significant effect on prescribing of antidepressants.

We thus do not find evidence that physicians prescribed their patients other drugs, whether to game the metric about which they were notified or to change the alignment of their prescribing to guidelines. These results are the same as those presented in eTable 4 in Supplement 2.

Patient Level Outcomes

Baseline patients were identified as those who had one or more quetiapine fills from study prescribers in the year before the letters were sent. Of the 103,758 such patients, we excluded those associated with multiple study prescribers (2.1%); those who were not enrolled in Medicare Part D in March 2015, immediately prior to the study (9.7%); and those who had died by the study start date (11.4%). Of the resulting 89,500 patients, 43,911 were aligned to the treatment arm and 45,589 were aligned to the control arm. See eFigure 1 of Supplement 2.

We classified 30,443 of the baseline patients as low-value, having Alzheimer's or dementia but not schizophrenia, bipolar disorder, or major depression. 24,628 were classified as guideline-concordant, having the psychiatric disorders without Alzheimer's or dementia. Using RxHCC (Part D risk-adjustment) codes based on 2013 and 2014 diagnosis codes, the psychiatric disorders were identified in patients by the presence of RxHCCs 58, 59, and 60; Alzheimer's and dementia were identified by RxHCCs 54 and 55. See eTable 1 of Supplement 2.

We did not pre-specify analyses of the intermediate group of baseline patients who fit neither the low-value nor the guideline-concordant definitions. This group comprised 40,330 patients. 16,858 of them had both a history of psychiatric disorders and a history of Alzheimer's or dementia, making the appropriate classification unclear. The remaining 23,472 lacked a diagnosis history of any of these disorders, also leaving the proper classification unclear.

Among those lacking a diagnosis history, 1,951 were new to Medicare and their diagnosis data was not populated for 2013 and 2014. The remaining 21,521 had been enrolled in Medicare long enough to have populated data in 2013 and/or 2014, but had no diagnoses that fit the low-value or guideline-concordant definitions.

The analysis plan described a second approach to identifying a patient cohort, using patients who received evaluation and management services from the study prescribers in the year prior to the intervention. Given the focus of the study on quetiapine prescribing and the multiplicity of outcomes already required by the analysis plan, this additional patient cohort was neither constructed nor analyzed.

The patients are described in more detail in eTable 2 in Supplement 2.

Patient Receipt of Quetiapine

A key question is how targeting the prescribers affects patients' source of quetiapine and their overall receipt of it. Even if study prescribers taper their own patients, they could refer them to

other prescribers, or patients could seek out other prescribers on their own volition. If these other prescribers have formal training in psychiatry they may have more knowledge of guidelines and recommendations about the prescribing of antipsychotics.

Table P7 explores the effect of the intervention on overall quetiapine receipt by the baseline patients as well as their receipt of quetiapine by source. In tallying quetiapine days for the outcome, column 1 counts prescriptions from all prescribers. Column 2 only counts prescriptions from the prescriber from which the patient received quetiapine in the year before the intervention – the patient's baseline prescriber. Column 3 considers prescriptions from all prescribers in Medicare Part D other than the baseline prescriber. Column 4 considers the subset of these prescriptions that were written by prescribers with psychiatric specialization according to their taxonomy codes in the NPPES database.

For each source the table displays, at the bottom, the contribution of that source to the total percent effect of the intervention on quetiapine receipt (i.e. the adjusted effect for the source divided by the control group all-prescriber average level of quetiapine receipt). It also displays the percent effect (i.e. the effect for the source divided by the control arm average level of quetiapine receipt from that source). By definition, these two effects are equal for the all-prescriber count (column 1) but are not equal for columns counting prescriptions from subsets of the prescribers (columns 2-4).

Baseline patients saw a 3.9% reduction in their receipt of quetiapine due to the intervention. The baseline study prescribers contributed 4.9 percentage points to this effect – that is, only counting the effect of the intervention on prescriptions from baseline study prescribers led to a 4.9% reduction in total quetiapine receipt. This reduction was offset by increased receipt from other prescribers; fills from these prescribers contributed a positive 1.0 percentage points to the effect. Of the 1.0 percentage points, 0.6 percentage points (i.e. three-fifths) of the effect came from psychiatric prescribers. All of these effects were statistically significant.

The contributions of the baseline prescribers and the other prescribers would sum to exactly the all-prescriber effect if the two regressions used the same control variables. However, the contributions are estimated from regressions that control for the outcome measured in the 9 months before the intervention began. The lagged outcome control is not identical across the regressions (i.e. prior receipt of quetiapine from the attributed prescriber does not equal prior receipt of quetiapine from other prescribers). Thus the estimated intervention effect on the components does not exactly sum to the estimated total intervention effect.

Quantile Effects

We prespecified analyses to estimate the effect of the intervention on points of the distribution of quetiapine receipt by patients. These analyses use quantile regressions. As in the prescriber-level analysis, we omit all control variables from the quantile regressions to ensure that we estimate unconditional effects.

Table P8 presents these results. Column 1 shows OLS results omitting controls to make the specification align with the subsequent quantile effects. This column shows that the intervention

reduced the average level of quetiapine receipt by 4.0%. Columns 2 through 6 show effects on the 10th, 25th, 50th, 75th and 90th quantiles.

A substantial fraction of the patients in the control group received no quetiapine in the 9 month post-intervention period; the 10th and 25th quantiles of the distribution were 0. The quantile effects at these points were 0 (columns 2 and 3); this is expected if the intervention reduces all points of the distribution since it cannot reduce receipt of quetiapine below zero. The intervention reduced the median by 6.2% (column 4). Columns 5 and 6 find no effect on the 75th and 90th quantiles, respectively. In the control arm, these quantiles correspond to a 270 day or more supply of quetiapine, sufficient for the entire 270 day (9 month) outcome horizon. These results suggest that the intervention did not reduce quetiapine receipt at the points in the distribution where patients had enough days supply of the drug to cover the entire outcome window.

Heterogeneous Effects on Patients

Table P9 shows the results looking at heterogeneity in effects for different groups of patients.

In columns 2 through 5 we divide patients into quartiles on the basis of their receipt of quetiapine in the 9 months prior to the intervention. We find that the effect of the intervention was strongest, in percent terms, for patients receiving less quetiapine before the letters were sent. The lowest quartile saw a 11.0% reduction in quetiapine, the second quartile saw a 5.2% reduction, and the third and fourth quartile saw 3.9% and 1.7% declines, respectively. In absolute terms, effects were largest for the third quartile (8.4 day reduction). The effects were statistically significant for all four quartiles.

The remaining columns split patients into two groups on the basis of whether they were previously associated with multiple prescribers or solely their baseline study prescriber. Specifically, column 6 limits to the 34% of baseline patients that received quetiapine from multiple prescribers – though, by definition of the patient cohort, only one study prescriber – in the 9 month pre-intervention period. The letters reduced quetiapine receipt by a statistically significant 1.9% for this group. Column 7 limits to the remaining 66% of patients that received quetiapine from only their attributed (i.e. study) prescriber during that time. The effect for these patients was a 5.2% reduction, almost three times larger than the effect for the patients who were not solely attached to their study prescriber.

These results show that patients who already had established links to other prescribers to receive quetiapine saw smaller reductions in quetiapine receipt due to the letters. While the findings are suggestive, these patients may have more easily moved to other prescribers to counteract reductions from study prescribers.

Effects for Low-Value and Guideline-Concordant Patients by Source

Table P10 shows the effects of the intervention on all-prescriber quetiapine receipt for low-value and guideline-concordant patients (columns 1 and 5, respectively). These effects were pre-specified in our analysis plan. For each category of patients it also breaks down the effect by quetiapine source (columns 2-4 and 6-8), as Table P7 did for the full set of baseline patients. These columns were not pre-specified and represent exploratory analyses. As in Table P7 and

explained in its supporting text, the effects of each source do not have to sum to exactly the all-prescriber effect. The table matches eTable 3 in Supplement 2 and Figure 3 in the main text, except that in this table we present the subdivision of other prescribers differently (showing a column for other non-psychiatric prescribers rather than a column for all other prescribers).

Low-value patients saw a statistically significant reduction in quetiapine receipt of 5.9%. 5.9 percentage points of this effect were due to the patients' baseline study prescribers, and this contribution was statistically significant. Other prescribers contributed 0.1 percentage points to this effect, and the contribution of the subset of these prescribers with psychiatric specialization was 0.0 percentage points. These contributions were not statistically significant.

The reduction for guideline-concordant patients was 2.4%. Baseline study prescribers contributed a reduction of 3.9 percentage points, offset by a positive contribution of 1.6 percentage points from other prescribers; the subset of these other prescribers with psychiatric specialization contributed 1.1 percentage points. All of these contributions were statistically significant.

Receipt of Other Psychiatric Drugs

For several reasons, patients may have changed their receipt of psychiatric drugs besides quetiapine as a result of the intervention. A patient's study prescriber may have shifted him or her to other drugs, the patient may have been referred to another prescriber who sought to treat the patient with a different approach, or the patients may have sought out other prescribers and in turn received other drugs.

Table P11 considers several categories of psychiatric drugs and finds no statistically effect on patient receipt of any of them. As in Table P6, which considered prescribers, we use the days supply of PDE rather than PDT so that the measurement is consistent across the categories (see Prescribing of Other Psychiatric Drugs on page 12 for a full description of the differences). This measure counts the days received (i.e. days supply dispensed to the patient) of drugs in the class. The first column of the table shows the quetiapine result of the main text using the days supply of PDE outcome rather than the baseline measure that uses PDT. We find a statistically significant reduction in quetiapine receipt of 3.9%, effectively the same result as the baseline measure.

Columns 2 and 3 consider atypical antipsychotics (other than quetiapine) and first generation antipsychotics. We estimate an increase in patient receipt of these classes of drugs of 2.9% and 4.0%, respectively; neither effect is statistically significant. The point estimate of 0.7 days for atypical antipsychotics would offset 11% of the 6.9 day reduction in quetiapine (the implied offset is 15% if the point estimate of 0.3 days for other antipsychotics is also included). Thus the overall reduction in quetiapine receipt is of similar magnitude even including effects on the receipt of other antipsychotics. For additional exploratory analyses of antipsychotic receipt by patient guideline conformity, see the main text and eTable 6 in Supplement 2.

Columns 4 through 7 consider, in order, benzodiazepine drugs often prescribed for psychiatric indications like anxiety, benzodiazepine drugs commonly prescribed as sleep aids, non-

benzodiazepine sleep aids, and antidepressants. We do not detect any statistically significant effects on patient receipt of these classes of drugs.

These results in Table P11 are the same as those presented in eTable 5 in Supplement 2.

Patient Mortality and Health Care Utilization

The impact of the intervention on patient health will depend on whether prescribing at the margin (and thus prescribing removed due to the intervention) was clinically beneficial or harmful. To provide more direct evidence on the quality of such marginal prescribing, we consider outcomes related to patient health: mortality and utilization of health care.

The Medicare administrative data includes information on the date of death of Medicare beneficiaries, allowing us to track mortality. We measure utilization through fee-for-service Medicare Part A and B claims, which include inpatient stays, emergency department encounters, and individual practitioner (including physicians, nurse practitioners, and other health care providers like psychologists) encounters. Because these claims are only available for Original Medicare enrollees, for utilization outcomes we reduce the sample of baseline patients to those enrolled in Original Medicare, both Parts A and B, in March 2015 (the baseline sample is already limited to those enrolled in Medicare Part D in March 2015). After this restriction, the baseline sample for utilization outcomes numbered 60,425 enrollees.

Table P12 presents the results. The outcome in column 1 is an indicator of patient death within 270 days of the intervention. We detect no effect on mortality.

The remaining columns look at different measures of claims-based care utilization in the 270 day outcome period. To avoid double counting when a provider bills for the same encounter over multiple claims, we treat multiple claims with the same provider on the same day as one encounter.

For inpatient and ED encounters, in addition to looking at total counts, we also subdivide them according to the underlying reason for the encounter using the patient's principal diagnosis code. We grouped the principal diagnosis codes into categories with the single-level index from Healthcare Cost and Utilization Project's (HCUP) Clinical Classifications Software (CCS). To identify principal diagnosis codes related to substance use disorder, we used the CCS categories 660 (alcohol-related disorders) and 661 (substance-related disorders). To identify principal diagnosis codes related to mental health, we followed the methodology of Heslin et al. (2015) and used CCS categories 650-652, 655-659, 662, 663, and 670.

Column 2 considers all inpatient visits, column 3 counts only the subset with a principal diagnosis of mental health conditions, and column 4 counts only the subset with a principal diagnosis of substance use disorders. Likewise, column 5 considers all ED encounters that did not end in an inpatient stay. Columns 6 and 7 tally the subsets of these encounters due to mental health conditions and substance use disorders, respectively. Although we find point estimates consistent with a decline in these encounters, particularly those due to mental health conditions, none of the estimates reach statistical significance.

Columns 8 and 9 analyze encounters with health care practitioners (we present two approaches in order to operationalize the prespecified outcome “receipt of outpatient mental health services”). Here we exclude encounters that occur in an inpatient or ED setting (specifically, those with a place of service of inpatient hospital, hospital emergency room, inpatient rehabilitation facility, or inpatient psychiatric facility) to focus on physician encounters apart from those due to utilization already measured in columns 2-7. Column 8 considers encounters with psychiatric providers: physicians with psychiatric specialization and psychiatric nurse practitioners. Column 9 considers encounters with providers carrying a specialization of psychologist. We detect no effect on either outcome.

As an exploratory exercise, eTable 7 in Supplement 2 presents these outcomes (without breaking out inpatient and ED encounters by principal diagnosis, and omitting psychologist encounters) for low-value and guideline-concordant patients separately, and is discussed in the main text.

Sensitivity and Robustness

The remaining tables test the robustness and sensitivity of the study outcomes.

Table P13 considers how the effects presented in Table 2 of the main text vary depending on the set of statistical controls. For each outcome, it presents the estimated absolute effect of the intervention under the three pre-specified approaches: using the full set of controls (“All Controls”), using the outcome measure in the 9 months before the intervention as a control (“Lag DV Control”), and using no controls (“Raw Difference”). The Lag DV Control approach for the patient-level columns includes controls for both the prior patient outcome and the prior attributed prescriber outcome.

In general, the lagged outcome variables have high explanatory power for the observed outcome, and including these variables as controls thus raises the statistical power of the regressions to detect effects. Consistent with this fact, the standard errors for prescriber level effects (columns 1-4) shrink approximately 30-60% with the inclusion of the lagged dependent variable; the standard errors for the patient level effects (columns 4-6) shrink approximately 20-40%. Adding further controls has little effect on the standard errors for prescriber level outcomes and yields a small additional reduction in standard errors (approximately 5-15%) for patient level outcomes.

The prescriber level effects (columns 1-4) attenuate and become more precisely estimated with the addition of the controls. The attenuation is nearly entirely from the addition of the lagged dependent variables. Patient level effect estimates (columns 5-7) become more precisely estimated with the addition of controls, and the point estimates here are less sensitive to the specification.

Table P14 shows how the effect on the primary outcome, days supply (of PDT), evolved over time by considering alternative measurement durations. The column headings show the exact number of days to which each duration corresponds. In the month after the letters were sent, we estimate that quetiapine prescribing declined in the treatment group by 5.4%. The magnitude of the decline increased to 11.1% at 9 months (the primary study outcome), to 12.8% at 1 year, to 14.7% at 18 months (the longest duration in the pre-specified analysis plan), and to 15.6% at 2 years (the exploratory extension of outcome duration reported in the main text).

Table P15 considers alternative approaches to measuring prescribing volume for the primary outcome. Column 1 replicates the main study measure (days supply of PDT). Column 2 measures prescribing in grams supplied of quetiapine. Column 3 measures it in prescription drug events (PDE) – the number of quetiapine fills associated with the prescriber, counting multiple fills to the same patient on the same day separately and not adjusting for the days supply on the fills. Column 4 measures the total days supply of the PDE (see Prescribing of Other Psychiatric Drugs on page 12 for a description of the difference between days supply of PDT and PDE). These approaches all yield similar estimates of prescribing declines. Column 5 measures the total Part D payments for the quetiapine fills, including payments made by beneficiaries and their Part D plans, and finds a larger decline of 15.4%.

References

American Geriatrics Society. 2015. “American Geriatrics Society 2015 Updated Beers Criteria for Potentially Inappropriate Medication Use in Older Adults.” *Journal of the American Geriatrics Society*. 63: 2227–2246.

Heslin, Kevin C, Elixhauser, Anne, Steiner, Claudia A. *Hospitalizations Involving Mental and Substance Use Disorders Among Adults, 2012*. Rockville, MD: Agency for Healthcare Research and Quality; 2015.

Lock Morgan, Kari and Donald B. Rubin. 2012. “Randomization to Improve Covariate Balance in Experiments.” *The Annals of Statistics*, 40(2): 1263-1282.

Powell, David. 2016. “Quantile Treatment Effects in the Presence of Covariates.” Unpublished manuscript available at https://works.bepress.com/david_powell/4/

Sacarny, Adam, David Yokum, Amy Finkelstein, and Shantanu Agrawal. 2016. “Medicare Letters To Curb Overprescribing Of Controlled Substances Had No Detectable Effect On Providers.” *Health Affairs (Millwood)*. 35(3): 471-479.

Tukey, John W. 1977. *Exploratory Data Analysis*. Reading, MA: Addison-Wesley.

Tables

Pre-Intervention Data

Table P1 - Summary Statistics about Prescribers

	(1) Control	(2) Treatment	(3) P-value
30-Day PDT, 2014 (average)	92.93	94.72	0.29
30-Day PDT, 2013 (average)	86.94	88.54	0.31
PDT (unadjusted), 2014 (average)	79.67	80.88	0.45
PDT (unadjusted), 2013 (average)	76.28	77.71	0.34
$ Payments, 2014 (average)	9,229	9,515	0.71
$ Payments, 2013 (average)	10,001	10,678	0.29
Census Northeast (share)	0.17	0.17	0.76
Census Midwest (share)	0.20	0.19	0.36
Census West (share)	0.18	0.17	0.29
Census South (share)	0.45	0.46	0.19
Contractor database (share)	[redacted]	[redacted]	0.44
Fraud Investigation Database (share)	[redacted]	[redacted]	0.11
Compromised Number Checklist (share)	[redacted]	[redacted]	0.31
Enrolled in PECOS (share)	0.69	0.71	0.22
Schedule II Study Outlier (share)	0.05	0.05	0.95
Joint test of equality P-value:			0.71
Prescribers	2,528	2,527	

See supplement for definition of PDT and 30-Day PDT. Total dollars paid refers to the total payments for the prescriptions, including payments from the Part D plan as well as out of pocket payments by the beneficiary. Column 3 lists p-values from t-tests that the mean of each variable is equal between treatment and control. Some shares are not displayed for confidentiality purposes. The joint test is the Wilks lambda F test for equality of means of the variables (excluding Share Census South, which is collinear with the other three census division shares).

Effects on Prescriber-Level Outcomes

Table P2 - Effect on New and Continuing Prescribing

	(1)	(2)	(3)	(4)	(5)
Approach		Lookback		Fill Field	
Patient Group	Baseline	New	Continuing	New	Continuing
N	5,055	5,055	5,055	5,055	5,055
Control Mean	2,863.97	218.74	2,645.23	1,157.12	1,706.84
Treatment Mean	2,455.82	157.19	2,298.63	960.65	1,495.17
Raw Difference	-408.14	-61.55	-346.59	-196.47	-211.67
	(71.49)	(6.59)	(66.52)	(34.50)	(43.98)
	[<0.001]	[<0.001]	[<0.001]	[<0.001]	[<0.001]
Adjusted Difference	-318.71	-59.26	-262.82	-173.81	-149.80
	(28.40)	(4.47)	(26.67)	(15.84)	(18.30)
	[<0.001]	[<0.001]	[<0.001]	[<0.001]	[<0.001]
Percent Difference	-11.13%	-27.09%	-9.94%	-15.02%	-8.78%
	(0.99%)	(2.04%)	(1.01%)	(1.37%)	(1.07%)
	[<0.001]	[<0.001]	[<0.001]	[<0.001]	[<0.001]

This table shows the effect of the intervention on prescribing to new patients and continuing patients. The lookback approach defines new patients as those who had not received a quetiapine prescription from the prescriber in the year prior to the fill; the fill field approach defines new patients as those making their initial fill on the prescription (i.e. non-refills). Fills not marked as new are counted as continuing. All outcomes consider fills in the 270 days after the intervention began. Robust standard errors in parentheses, p-values in brackets.

Table P3 - Effect on Characteristics of the Average Patient

Characteristic	(1)<br>Age	(2)<br>Institutional-ized	(3)<br>Hx Dementia	(4)<br>Hx Maj Psych
N	5,055	5,055	5,055	5,055
Control Mean	69.784	0.097	0.378	0.436
Treatment Mean	69.888	0.098	0.380	0.447
Raw Difference	0.104	0.002	0.002	0.010
	(0.291)	(0.005)	(0.008)	(0.007)
	[0.721]	[0.713]	[0.819]	[0.143]
Adjusted Difference	-0.021	0.005	0.003	0.009
	(0.108)	(0.002)	(0.003)	(0.004)
	[0.845]	[0.003]	[0.345]	[0.012]
Percent Difference	-0.03%	5.68%	0.84%	2.04%
	(0.16%)	(1.92%)	(0.89%)	(0.81%)
	[0.845]	[0.003]	[0.345]	[0.012]

This table shows the effect of the intervention on the characteristics of the average patient treated by the prescribers. The regression outcome is the average of the characteristic across the treatments attributed to the prescriber in the 270 days after the intervention began. Institutionalized indicates residing in long term institution in March 2015. Hx Dementia is a dementia or Alzheimer's diagnosis in 2013 or 2014 (RxHCCs 54 or 55). Hx Maj Psych is a schizophrenia, bipolar disorder, or major depression diagnosis in 2013 or 2014 (RxHCCs 58, 59, or 60). Robust standard errors in parentheses, p-values in brackets.

Table P4 - Heterogeneous Effects

	(1)	(2)	(3)	(4)	(5)	(6)	(7)	(8)	(9)
		Previous Fraud Investig		Ex Ante Rx Volume		Ex Ante Rx Volume (Quartiles)*			
Prescriber Group	Baseline	No Record	≥1 Record	< Median	> Median	Quartile 1	Quartile 2	Quartile 3	Quartile 4
N	5,055	4,448	607	2,527	2,528	1,264	1,263	1,268	1,260
Control Mean	2,863.97	2,684.66	4,242.37	1,421.75	4,354.92	1,083.09	1,778.26	2,641.78	5,948.35
Treatment Mean	2,455.82	2,318.31	3,418.00	1,335.77	3,538.40	1,007.46	1,647.58	2,315.57	4,866.44
Raw Difference	-408.14	-366.35	-824.37	-85.98	-816.52	-75.63	-130.68	-326.21	-1081.91
	(71.49)	(60.84)	(383.58)	(26.73)	(120.37)	(30.67)	(34.81)	(48.22)	(204.26)
	[<0.001]	[<0.001]	[0.032]	[0.001]	[<0.001]	[0.014]	[<0.001]	[<0.001]	[<0.001]
Adjusted Difference	-318.71	-279.98	-624.12	-126.12	-520.69	-106.03	-145.43	-325.82	-707.07
	(28.40)	(25.88)	(132.36)	(20.81)	(54.76)	(25.80)	(32.66)	(44.49)	(99.48)
	[<0.001]	[<0.001]	[<0.001]	[<0.001]	[<0.001]	[<0.001]	[<0.001]	[<0.001]	[<0.001]
Percent Difference	-11.13%	-10.43%	-14.71%	-8.87%	-11.96%	-9.79%	-8.18%	-12.33%	-11.89%
	(0.99%)	(0.96%)	(3.12%)	(1.46%)	(1.26%)	(2.38%)	(1.84%)	(1.68%)	(1.67%)
	[<0.001]	[<0.001]	[<0.001]	[<0.001]	[<0.001]	[<0.001]	[<0.001]	[<0.001]	[<0.001]

This table shows the effect of the intervention for different prescriber subgroups. Columns 2 and 3 split the prescribers depending on whether they had at least 1 record in the Fraud Investigation Database, Compromised Number Checklist or the Medicare Part D integrity contractor's database. Columns 4 and 5 split the prescribers depending on whether their prescribing in the 9 months prior to the intervention was above or below the median. Columns 6-9 divide the prescribers by quartile of their prescribing in the 9 months prior to the intervention. All outcomes consider fills in the 270 days after the intervention began. Robust standard errors in parentheses, p-values in brackets.

* Exploratory analysis (not pre-specified).

Table P5 - Quantile Treatment Effects

	(1)	(2)	(3)	(4)	(5)	(6)
Regression Method	OLS	Quantile Regression				
Statistic	Mean	q10	q25	q50	q75	q90
N	5,055	5,055	5,055	5,055	5,055	5,055
Control Mean or Quantile	2,863.97	780.00	1,350.00	2,146.00	3,395.00	5,644.00
Treatment Mean or Quantile	2,455.82	750.00	1,230.00	1,924.00	3,002.00	4,629.00
Raw Difference	-408.14	-30.00	-120.00	-222.00	-393.00	-1,015.00
	(71.49)	(43.96)	(34.96)	(49.08)	(96.59)	(197.42)
	[<0.001]	[0.495]	[<0.001]	[<0.001]	[<0.001]	[<0.001]
Percent Difference	-14.25%	-3.85%	-8.89%	-10.34%	-11.58%	-17.98%
	(2.50%)	(5.64%)	(2.59%)	(2.29%)	(2.85%)	(3.50%)
	[<0.001]	[0.495]	[<0.001]	[<0.001]	[<0.001]	[<0.001]

This table shows the effect of the intervention on the distribution of prescribing volume (i.e. the primary study outcome). All regressions do not include control variables because these variables change the interpretation of the quantile regressions. Column 1 uses linear multivariable regression (ordinary least squares) to estimate the effect of the intervention on the mean, replicating the main study results, albeit without any control variables. Columns 2-6 use quantile regression to estimate quantile treatment effects -- the effect of the intervention on the quantiles. The percent difference is the raw difference divided by the control group mean or quantile. All outcomes consider fills in the 270 days after the intervention began. Robust standard errors in parentheses, p-values in brackets.

Table P6 - Effect on Prescribing of Other Psychiatric Drugs

	(1)	(2)	(3)	(4)	(5)	(6)	(7)
	Antipsychotics			Benzodiazepines		Non-BZD	Anti-
Drug	Quetiapine	Oth Atypical	1st Gen	Psych	Sleep	Sleep	Depressants
N	5,055	5,055	5,055	5,055	5,055	5,055	5,055
Control Mean	2,994.34	2,771.61	554.25	11,254.08	1,406.45	2,645.77	18,900.46
Treatment Mean	2,561.18	2,416.70	472.34	11,322.43	1,446.75	2,606.55	18,381.58
Raw Difference	-433.16	-354.91	-81.90	68.36	40.30	-39.22	-518.88
	(76.66)	(109.68)	(30.48)	(290.24)	(69.79)	(68.65)	(383.52)
	[<0.001]	[0.001]	[0.007]	[0.814]	[0.564]	[0.568]	[0.176]
Adjusted Difference	-330.39	-45.15	-15.52	-76.58	10.64	-30.75	-12.91
	(30.11)	(30.26)	(9.86)	(83.12)	(22.41)	(24.19)	(96.99)
	[<0.001]	[0.136]	[0.115]	[0.357]	[0.635]	[0.204]	[0.894]
Percent Difference	-11.03%	-1.63%	-2.80%	-0.68%	0.76%	-1.16%	-0.07%
	(1.01%)	(1.09%)	(1.78%)	(0.74%)	(1.59%)	(0.91%)	(0.51%)
	[<0.001]	[0.136]	[0.115]	[0.357]	[0.635]	[0.204]	[0.894]

This table shows the effect of the intervention on the prescribing of quetiapine and other psychiatric drugs. All outcomes are measured in the total days supply of PDE, rather than days supply of PDT used elsewhere, so that the units are common across the drug categories (see text). Column 1 shows the days supply of quetiapine, column 2 considers atypical antipsychotics excluding quetiapine, and column 3 considers first generation antipsychotics. Columns 4 and 5 consider benzodiazepines commonly prescribed for psychiatic and sleep indications, respectively. Column 6 counts non-benzodiazepine sleep aids, and column 7 counts antidepressants. All outcomes consider fills in the 270 days after the intervention began. Robust standard errors in parentheses, p-values in brackets.

Effects on Patient-Level Outcomes

Table P7 - Patient Receipt of Quetiapine by Source

Source	(1) All Prescribers	(2) Baseline Prescriber	(3) Other Prescribers	(4)
			All Others	Psych Only
N	89,500	89,500	89,500	89,500
Control Mean	169.69	119.58	50.11	13.77
Treatment Mean	162.93	111.73	51.20	14.38
Raw Difference	-6.76	-7.85	1.09	0.62
	(1.80)	(1.73)	(1.22)	(0.63)
	[<0.001]	[<0.001]	[0.369]	[0.326]
Adjusted Difference	-6.66	-8.31	1.71	0.95
	(0.92)	(1.05)	(0.77)	(0.35)
	[<0.001]	[<0.001]	[0.025]	[0.007]
Percent Difference	-3.92%	-6.95%	3.41%	6.92%
	(0.54%)	(0.88%)	(1.53%)	(2.58%)
	[<0.001]	[<0.001]	[0.025]	[0.007]
Contribution to Total Percent Difference	-3.92%	-4.90%	1.01%	0.56%
	(0.54%)	(0.62%)	(0.45%)	(0.21%)
	[<0.001]	[<0.001]	[0.025]	[0.007]

This table shows the effect of the intervention on baseline patient receipt of quetiapine broken down by source. Column 1 counts days supplied from all prescribers. Column 2 counts only days from the patient's baseline prescriber (i.e. prescriber from whom the patient received quetiapine in the baseline period). Column 3 counts days from all prescribers other than the baseline one, and column 4 looks only at those other prescribers with a psychiatric specialization. The contribution to the total percent difference is defined as the adjusted difference divided by the all-prescriber (column 1) control mean. All outcomes consider fills in the 270 days after the intervention began. Standard errors clustered at the baseline prescriber level in parentheses, p-values in brackets.

Table P8 - Quantile Treatment Effects for Patient Receipt of Quetiapine

	(1)	(2)	(3)	(4)	(5)	(6)
Regression Method	OLS	Quantile Regression				
Statistic	Mean	q10	q25	q50	q75	q90
N	89,500	89,500	89,500	89,500	89,500	89,500
Control Mean or Quantile	169.69	0.00	0.00	192.00	270.00	300.00
Treatment Mean or Quantile	162.93	0.00	0.00	180.00	270.00	300.00
Raw Difference	-6.76	0.00	0.00	-12.00	0.00	0.00
	(1.80)	(0.64)	(0.45)	(12.03)	(0.48)	(0.76)
	[<0.001]	[1.000]	[1.000]	[0.318]	[1.000]	[1.000]
Percent Difference	-3.98%	N/A	N/A	-6.25%	0.00%	0.00%
	(1.06%)			(6.26%)	(0.18%)	(0.25%)
	[<0.001]			[0.318]	[1.000]	[1.000]

This table shows the effect of the intervention on the distribution of baseline patient receipt of quetiapine. All regressions do not include control variables because these variables change the interpretation of the quantile regressions. Column 1 uses linear multivariable regression (ordinary least squares) to estimate the effect of the intervention on the mean, replicating the main study results, albeit without any controls. Columns 2-6 use quantile regression to estimate quantile treatment effects -- the effect of the intervention on the quantiles. The percent difference is the raw difference divided by the control group mean or quantile. All outcomes consider fills in the 270 days after the intervention began. Standard errors clustered at the baseline prescriber level in parentheses, p-values in brackets.

Table P9 - Heterogeneous Effects for Patients

	(1)	(2)	(3)	(4)	(5)	(6)	(7)
		Prior Quetiapine Receipt Volume (Quartiles)				Share from Baseline Rx'er	
Patient Group	Baseline	Quartile 1	Quartile 2	Quartile 3	Quartile 4	<100%	100%
N	89,500	19,003	21,540	31,775	17,182	30,435	59,065
Control Mean	169.69	48.96	130.67	216.34	265.25	196.80	155.51
Treatment Mean	162.93	44.70	123.21	208.44	260.03	193.43	147.47
Raw Difference	-6.76	-4.26	-7.46	-7.91	-5.23	-3.37	-8.04
	(1.80)	(1.96)	(1.91)	(1.47)	(2.30)	(2.19)	(1.98)
	[<0.001]	[0.029]	[<0.001]	[<0.001]	[0.023]	[0.124]	[<0.001]
Adjusted Difference	-6.66	-5.37	-6.80	-8.40	-4.43	-3.81	-8.06
	(0.92)	(1.47)	(1.73)	(1.34)	(1.93)	(1.40)	(1.08)
	[<0.001]	[<0.001]	[<0.001]	[<0.001]	[0.022]	[0.007]	[<0.001]
Percent Difference	-3.92%	-10.96%	-5.20%	-3.88%	-1.67%	-1.93%	-5.18%
	(0.54%)	(2.99%)	(1.33%)	(0.62%)	(0.73%)	(0.71%)	(0.69%)
	[<0.001]	[<0.001]	[<0.001]	[<0.001]	[0.022]	[0.007]	[<0.001]

This table shows the effect of the intervention for different patient subgroups. Columns 2 through 5 divide the patients by quartile of their quetiapine receipt in the 9 months prior to the intervention. Columns 6 considers patients who received quetiapine both from their baseline study prescriber and at least one other prescriber in the 12 months before the intervention; column 7 considers patients who previously only received quetiapine from their baseline study prescriber. All outcomes consider fills in the 270 days after the intervention began. Standard errors clustered at the baseline prescriber level in parentheses, p-values in brackets.

Table P10 - Effect on Source of Quetiapine by Guideline Classification

Source	(1)	(2)	(3)	(4)	(5)	(6)	(7)	(8)
	Low-Value				Guideline-Concordant			
	All Prescribers	Baseline Prescriber*	Other Prescribers*		All Prescribers	Baseline Prescriber*	Other Prescribers*	
			All Others	Psych Only			All Others	Psych Only
N	23,490	23,490	23,490	23,490	25,680	25,680	25,680	25,680
Control Mean	158.75	116.49	42.26	4.46	182.14	115.65	66.49	28.55
Treatment Mean	147.87	105.80	42.07	4.54	177.86	109.45	68.41	29.37
Raw Difference	-10.88	-10.69	-0.19	0.08	-4.29	-6.21	1.92	0.83
	(2.12)	(2.20)	(1.76)	(0.49)	(2.62)	(2.54)	(2.06)	(1.41)
	[<0.001]	[<0.001]	[0.912]	[0.862]	[0.102]	[0.014]	[0.350]	[0.558]
Adjusted Difference	-9.43	-9.38	-0.11	0.02	-4.46	-7.18	2.85	1.92
	(1.64)	(1.73)	(1.24)	(0.37)	(1.41)	(1.56)	(1.26)	(0.84)
	[<0.001]	[<0.001]	[0.928]	[0.968]	[0.002]	[<0.001]	[0.023]	[0.022]
Percent Difference	-5.94%	-8.05%	-0.27%	0.34%	-2.45%	-6.21%	4.29%	6.72%
	(1.03%)	(1.49%)	(2.94%)	(8.34%)	(0.77%)	(1.35%)	(1.89%)	(2.93%)
	[<0.001]	[<0.001]	[0.928]	[0.968]	[0.002]	[<0.001]	[0.023]	[0.022]
Contribution to Total	-5.94%	-5.91%	-0.07%	0.01%	-2.45%	-3.94%	1.57%	1.05%
Percent Difference	(1.03%)	(1.09%)	(0.78%)	(0.23%)	(0.77%)	(0.86%)	(0.69%)	(0.46%)
	[<0.001]	[<0.001]	[0.928]	[0.968]	[0.002]	[<0.001]	[0.023]	[0.022]

This table shows the effect of the intervention on patients' source of quetiapine, split by guideline appropriateness for the drug. Columns 1-4 consider only low-value patients while columns 5-8 consider only guideline-concordant patients. See notes for Table P7 for definitions of the source. The contribution to the total percent difference is defined as the adjusted difference divided by the all-prescriber (column 1 or 5) control mean. All outcomes consider fills in the 270 days after the intervention began. Standard errors clustered at the baseline prescriber level in parentheses, p-values in brackets.

* Exploratory analysis (not pre-specified).

Table P11 - Effect on Patient Receipt of Other Psychiatric Drugs

	(1)	(2)	(3)	(4)	(5)	(6)	(7)
	Antipsychotics			Benzodiazepines		Non-BZD	Anti-
Drug	Quetiapine	Oth Atypical	1st Gen	Psych	Sleep	Sleep	Depressants
N	89,500	89,500	89,500	89,500	89,500	89,500	89,500
Control Mean	178.69	25.83	7.47	75.83	8.09	12.79	119.13
Treatment Mean	171.07	25.88	6.85	76.55	8.65	13.52	116.82
Raw Difference	-7.61	0.05	-0.62	0.72	0.57	0.74	-2.31
	(1.99)	(0.91)	(0.39)	(1.41)	(0.54)	(0.47)	(1.49)
	[<0.001]	[0.955]	[0.116]	[0.611]	[0.291]	[0.118]	[0.121]
Adjusted Difference	-6.93	0.74	0.30	0.28	0.02	0.27	0.45
	(0.98)	(0.48)	(0.18)	(0.49)	(0.20)	(0.23)	(0.65)
	[<0.001]	[0.125]	[0.099]	[0.574]	[0.907]	[0.246]	[0.483]
Percent Difference	-3.88%	2.85%	4.03%	0.37%	0.28%	2.12%	0.38%
	(0.55%)	(1.86%)	(2.45%)	(0.65%)	(2.44%)	(1.83%)	(0.54%)
	[<0.001]	[0.125]	[0.099]	[0.574]	[0.907]	[0.246]	[0.483]

This table shows the effect of the intervention on patients' receipt of quetiapine and other psychiatric drugs. All outcomes are measured in the total days supply of PDE, rather than days supply of PDT used elsewhere, so that the units are common across the drug categories (see text). Column 1 shows the days supply of quetiapine, column 2 considers atypical antipsychotics excluding quetiapine, and column 3 considers first generation antipsychotics. Columns 4 and 5 consider benzodiazepines commonly prescribed for psychiatic and sleep indications, respectively. Column 6 counts non-benzodiazepine sleep aids, and column 7 counts antidepressants. All outcomes consider fills in the 270 days after the intervention began. Standard errors clustered at the baseline prescriber level in parentheses, p-values in brackets.

Table P12 - Effect on Patient Mortality and Health Care Utilization

	(1)	(2)	(3)	(4)	(5)	(6)	(7)	(8)	(9)
		Inpatient Stays			ED Encounters				
Outcome	Death	All	Substance	Mental	All	Substance	Mental	Psychiatric	Psychologist
N	89,500	60,425	60,425	60,425	60,425	60,425	60,425	60,425	60,425
Control Mean	0.104	0.528	0.014	0.068	0.854	0.018	0.049	0.791	0.491
Treatment Mean	0.100	0.509	0.013	0.060	0.867	0.014	0.042	0.779	0.462
Raw Difference	-0.004	-0.018	0.000	-0.008	0.013	-0.004	-0.006	-0.012	-0.029
	(0.003)	(0.013)	(0.002)	(0.005)	(0.024)	(0.003)	(0.004)	(0.041)	(0.050)
	[0.248]	[0.156]	[0.863]	[0.108]	[0.578]	[0.197]	[0.091]	[0.763]	[0.559]
Adjusted Difference	-0.002	-0.015	0.000	-0.005	-0.008	-0.003	-0.005	-0.010	-0.012
	(0.002)	(0.010)	(0.002)	(0.003)	(0.015)	(0.003)	(0.003)	(0.021)	(0.024)
	[0.411]	[0.132]	[0.857]	[0.099]	[0.605]	[0.203]	[0.070]	[0.625]	[0.607]
Percent Difference	-1.66%	-2.79%	-1.99%	-8.06%	-0.93%	-17.84%	-10.57%	-1.29%	-2.47%
	(2.02%)	(1.85%)	(11.04%)	(4.89%)	(1.80%)	(14.02%)	(5.83%)	(2.63%)	(4.80%)
	[0.411]	[0.132]	[0.857]	[0.099]	[0.605]	[0.203]	[0.070]	[0.625]	[0.607]

This table shows the effect of the intervention on patients' mortality and health care utilization. Column 1 uses an indicator for mortality within 270 days of the intervention as the outcome. This column uses the main study sample. The utilization outcomes (columns 2-9) use the subset of main study sample that was enrolled in Original Medicare (Parts A and B) in March 2015. The outcomes are the number of provider encounters in the 270 days after the intervention began. Multiple encounters with the same provider on the same day are only counted as one encounter. Columns 2-4 count inpatient stays. Columns 5-7 count emergency department encounters that did not result in hospitalization. Inpatient and ED encounters are analyzed separately by principal diagnosis: any (columns 2 and 5), substance use disorder (columns 3 and 6), and mental health (columns 4 and 7). Column 8 counts encounters with psychiatric providers (physicians with psychiatric specialization and psychiatric nurse practitioners). Column 9 counts encounters with psychologists. Standard errors clustered at the baseline prescriber level in parentheses, p-values in brackets.

Sensitivity and Robustness Tests

Table P13 - Sensitivity of Study Outcomes to Statistical Controls

	(1)	(2)	(3)	(4)	(5)	(6)	(7)
Unit of Analysis	Prescribers				Patients		
Subset	To All	To New Patients	To Low-Value Patients	To Guideline-Concordant Patients	All Patients	Low-Value Patients	Guideline-Concordant Patients
N	5,055	5,055	5,055	5,055	89,500	23,490	25,680
Control Mean	2,863.97	218.74	752.88	753.16	169.69	158.75	182.14
Treatment Mean	2,455.82	157.19	619.01	664.86	162.93	147.87	177.86
Raw Difference	-408.14	-61.55	-133.87	-88.30	-6.76	-10.88	-4.29
	(71.49)	(6.59)	(31.85)	(23.69)	(1.80)	(2.12)	(2.62)
	[<0.001]	[<0.001]	[<0.001]	[<0.001]	[<0.001]	[<0.001]	[0.102]
Adjusted Difference,	-327.37	-58.83	-91.48	-74.14	-6.81	-9.80	-4.76
Lag DV Control	(28.63)	(4.49)	(12.46)	(10.64)	(1.06)	(1.73)	(1.56)
	[<0.001]	[<0.001]	[<0.001]	[<0.001]	[<0.001]	[<0.001]	[0.002]
Adjusted Difference,	-318.71	-59.26	-91.02	-73.90	-6.66	-9.43	-4.46
All Controls	(28.40)	(4.47)	(12.27)	(10.59)	(0.92)	(1.64)	(1.41)
	[<0.001]	[<0.001]	[<0.001]	[<0.001]	[<0.001]	[<0.001]	[0.002]

This table shows the sensitivity of the study outcomes reported in Table 2 of the main text to the use of statistical controls. The "Adjusted Difference, All Controls" specification is reported in the main text. The "Adjusted Difference, Lag DV Control" specification is only reported here. In columns 1-4 it uses as a control the prescriber's outcome variable in the 270 days before the intervention began (total quetiapine days supplied). The patient-level regressions (columns 5-7) use the baseline prescriber's lagged outcome variable (total quetiapine days supplied) and, additionally, the patient's lagged outcome variable. The "Raw Difference" specification is reported in the main text and uses no control variables. Robust standard errors in parentheses. In columns 5-7, standard errors are clustered at the baseline prescriber level. P-values in brackets.

Table P14 - Sensitivity of Primary Outcome to Duration

Duration	(1)<br>1 Month<br>30 Days	(2)<br>3 Months<br>90 Days	(3)<br>6 Months<br>180 Days	(4)<br>9 Months<br>270 Days	(5)<br>1 Year<br>365 Days	(6)<br>15 Months<br>455 Days	(7)<br>18 Months<br>545 Days	(8)<br>21 Months<br>635 Days*	(9)<br>2 Years<br>730 Days*
N	5,055	5,055	5,055	5,055	5,055	5,055	5,055	5,055	5,055
Control Mean	328.29	957.31	1,915.63	2,863.97	3,838.71	4,749.92	5,652.89	6,534.92	7,435.73
Treatment Mean	300.20	861.90	1,687.83	2,455.82	3,226.51	3,938.17	4,643.94	5,337.69	6,052.01
Raw Difference	-28.08	-95.41	-227.80	-408.14	-612.20	-811.75	-1,008.95	-1,197.23	-1,383.71
	(8.39)	(23.73)	(47.79)	(71.49)	(95.91)	(119.09)	(142.47)	(165.45)	(188.15)
	[<0.001]	[<0.001]	[<0.001]	[<0.001]	[<0.001]	[<0.001]	[<0.001]	[<0.001]	[<0.001]
Adjusted Difference	-17.75	-62.53	-165.52	-318.71	-490.52	-662.34	-831.64	-993.84	-1,156.53
	(3.59)	(7.05)	(16.66)	(28.40)	(42.93)	(55.70)	(68.65)	(82.08)	(95.28)
	[<0.001]	[<0.001]	[<0.001]	[<0.001]	[<0.001]	[<0.001]	[<0.001]	[<0.001]	[<0.001]
Percent Difference	-5.41%	-6.53%	-8.64%	-11.13%	-12.78%	-13.94%	-14.71%	-15.21%	-15.55%
	(1.09%)	(0.74%)	(0.87%)	(0.99%)	(1.12%)	(1.17%)	(1.21%)	(1.26%)	(1.28%)
	[<0.001]	[<0.001]	[<0.001]	[<0.001]	[<0.001]	[<0.001]	[<0.001]	[<0.001]	[<0.001]

This table shows the sensitivity of the primary outcome to different measurement durations. Table 2 of the main text reports the 270 days (column 4) and 730 days (column 9) results. Robust standard errors in parentheses, p-values in brackets.

* Exploratory analysis (not pre-specified).

Table P15 - Effect on Total Quetiapine Prescribing: Alternative Measures

Measure	(1)<br>Days Supply<br>(of PDT)	(2)<br><br>Grams	(3)<br><br>PDE	(4)<br>Days Supply<br>(of PDE)	(5)<br><br>$ Payments
N	5,055	5,055	5,055	5,055	5,055
Control Mean	2,863.97	423.77	93.97	2,994.34	6,377.32
Treatment Mean	2,455.82	375.71	79.04	2,561.18	5,539.80
Raw Difference	-408.14	-48.06	-14.93	-433.16	-837.52
	(71.49)	(12.31)	(2.89)	(76.66)	(312.36)
	[<0.001]	[<0.001]	[<0.001]	[<0.001]	[0.007]
Adjusted Difference	-318.71	-44.14	-10.15	-330.39	-979.13
	(28.40)	(5.78)	(1.04)	(30.11)	(174.73)
	[<0.001]	[<0.001]	[<0.001]	[<0.001]	[<0.001]
Percent Difference	-11.13%	-10.42%	-10.80%	-11.03%	-15.35%
	(0.99%)	(1.36%)	(1.11%)	(1.01%)	(2.74%)
	[<0.001]	[<0.001]	[<0.001]	[<0.001]	[<0.001]

This table shows the sensitivity of the primary outcome to different measures of quetiapine prescribing volume. Column 1 shows the days supply of prescription drug treatments (PDT), the primary study outcome. Column 2 shows grams of quetiapine dispensed. Column 3 shows the number of prescription drug events (PDE), or prescription fills. Column 4 shows the days supply of PDE. Column 5 shows total Medicare Part D payments for the fills. All outcomes consider fills in the 270 days after the intervention began. Robust standard errors in parentheses, p-values in brackets.

Attachments

Attachment 1 (Placebo Letter)

Department of Health & Human Services
7500 Security Boulevard, Mail Stop AR-18-50
Baltimore, Maryland 21244-1850

CMS
CENTERS FOR MEDICARE & MEDICAID SERVICES
CENTER FOR PROGRAM INTEGRITY

April 20, 2015

Pat Q. Provider MD
1234 Main St
Columbia, MD 21045
NPI: 1234567890
Specialty: General Care Practitioner

Re: Provider Enrollment Needed for Writing Prescriptions for Medicare Part D Drugs

The Centers for Medicare & Medicaid Services (CMS) recently finalized a new rule that requires physicians and other eligible professionals who write prescriptions for Part D drugs to be enrolled in an approved status or to have a valid opt-out affidavit on file for their prescriptions to be covered under Part D. The enclosed Medicare Learning Network® (MLN) Matters® article offers more information about the rule change and how it may affect you.

If you write prescriptions for covered Part D drugs and you are not enrolled in Medicare in an approved status or have a valid record of opting out, you need to submit an enrollment application or an opt out affidavit to your Medicare Administrative Contractor (MAC) by June 1, 2015, or earlier. Please consult the MLN Matters® document for guidance on how to submit and verify your enrollment.

Sincerely,

[redacted]

Investigations and Audit Group

Attachment 2 (Placebo Correction Letter)

DEPARTMENT OF HEALTH & HUMAN SERVICES
CENTERS for MEDICARE & MEDICAID SERVICES
7500 Security Boulevard, Mail Stop AR-18-50
Baltimore, Maryland 21244-1850

CMS
CENTERS FOR MEDICARE & MEDICAID SERVICES
CENTER FOR PROGRAM INTEGRITY

Investigations & Audits Group

June 10, 2015

[redacted]

NPI: [redacted]
Specialty: General Care Practitioner

Re: Provider Enrollment Needed for Writing Prescriptions for Medicare Part D Drugs

You recently received a letter dated April 20, 2015 that stated the Centers for Medicare & Medicaid Services (CMS) has implemented a new rule requiring physicians and other eligible professionals who write prescriptions for Medicare Part D drugs to be enrolled in Medicare in an approved status or have validly opted out for their prescriptions to be covered under Part D. CMS received multiple inquiries from providers questioning the intent of the letter. This notice seeks to address those questions.

It is important to note that if you are currently enrolled in Medicare in an approved status or have validly opted out, no action is required. To verify your enrollment status, CMS has made available an enrollment file that identifies a list of physicians and eligible professionals who are enrolled in Medicare in an approved or opt out status and are eligible to prescribe. The enrollment file is available at https://data.cms.gov/dataset/Medicare-Individual-Provider-List/u8u9-2upx on the CMS website. If you write prescriptions for covered Part D drugs and you are not identified on the enrollment file, you need to submit an enrollment application or an opt out affidavit to your Medicare administrative Contractor (MAC).

For more information on the Part D prescriber enrollment requirements, refer to MLN Matters® article SE1434 located at http://www.cms.gov/Outreach-and-Education/Medicare-Learning-Network-MLN/MLNMattersArticles/downloads/SE1434.pdf. Please note that an interim final rule is currently open to comments and proposes to change the enforcement date by which prescription drug claims will be denied by the Part D plans if the prescriber is not enrolled in Medicare in an approved status or validly opted-out to January 1, 2016. For more information see https://www.federalregister.gov/public-inspection. If you have questions regarding prescriber enrollment, please email CMS at providerenrollment@cms.hhs.gov.

Sincerely,

[redacted]

Investigations and Audits Group

Attachment 3 (Treatment Letter)

Department of Health & Human Services
7500 Security Boulevard, Mail Stop AR-18-50
Baltimore, Maryland 21244-1850

CENTERS FOR MEDICARE & MEDICAID SERVICES
CENTER FOR PROGRAM INTEGRITY

April 20, 2015

Pat Q. Provider MD
1234 Main St
Columbia, MD 21045
NPI: 1234567890 / Specialty: General Care Practitioner

Re: Your Seroquel prescribing is under review by the Center for Program Integrity.

Dear Dr. Provider,

The figure to the right displays your prescribing of Seroquel treatments (Seroquel, Seroquel XR, or generic quetiapine) compared to other general care practitioners in Maryland.

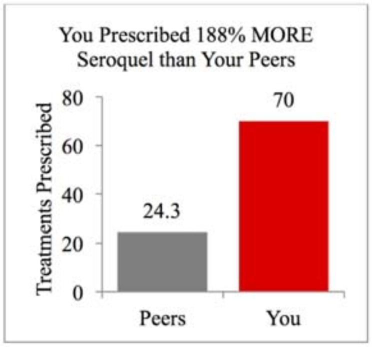

As can be seen, **you prescribed far more treatments – 188% more – than similar prescribers within your state.** In turn, you have been flagged as a markedly unusual prescriber, subject to review by the Center for Program Integrity.

We recognize that some flagged practitioners have appropriate reasons for this pattern. However, we have seen that other practitioners may drift into prescribing patterns that would be considered medically unjustified or abusive. Abusive prescribing can lead to extensive audits and even revocation of Medicare billing privileges.

We hope that you will use this information to see if your high prescribing level is consistent with the latest standards of care. To assist in your monitoring efforts, CMS will periodically send you letters with our most recent information about your Seroquel prescribing. **We may contact you at a later date to ask what steps, if any, you have taken in response to our communications.**

Read on for more information about the methodology used to analyze your prescribing behavior and to learn what actions to take next.

Sincerely,

[redacted]

Investigations and Audit Group

Introduction

Prescribers and pharmacies have a frontline role in assisting the Centers for Medicare & Medicaid Services (CMS) to effectively manage Medicare resources and monitor prescribing practices. CMS and its partners acknowledge the daily challenges prescribers and pharmacies face in serving Medicare beneficiaries and the complexity of billing for prescription drugs.

The Office of Inspector General (OIG) released a study in June 2013 showing over 1 million individual prescribers ordered drugs paid by Medicare Part D in 2009. Prescribing patterns varied widely by type of prescriber. Over 700 general-care physicians had questionable prescribing patterns.[1] Although some of this prescribing may be appropriate, the OIG's study expressed the need to further scrutinize such questionable patterns.

Using a similar methodology to that used in the OIG study, CMS analyzed Seroquel, Seroquel XR, and generic quetiapine prescribing data for calendar year 2013 and 2014.[2] Based on this analysis, CMS has determined that the number and quantity of your treatments exceeded the established threshold (see box on right). The intent of this letter is to inform you of the extent of your potential outlier status, which reflects the Seroquel treatments attributed to your prescribing practice compared to your peers within your prescriber type (general care practitioner) and state (Maryland).

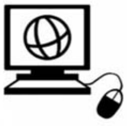

More Information

To learn more about Seroquel prescribing and to find details on our methodology, visit (this link is case sensitive): http://go.cms.gov/CPImethodologyhr2

We hope you find this information helpful and that it will provide insights into your current and future prescribing practices. We also hope that you will use the information provided to see if your high prescribing level for Seroquel is appropriate for your patient population.

Your Results

Table 1: Values Used to Determine Your Status as a Potential Outlier

Categories	Total Treatments	30-Day Equivalent
Your Values in 2014	70	79
Average of General Care Practitioners in Maryland	24.3	26.7

This table reviews the data that led to your classification as a potential outlier.

[1] OIG, Prescribers with Questionable Patterns in Medicare Part D, OEI-02-09-00603, June 2013

[2] The prescribing data consists of Prescription Drug Event (PDE) records. Each PDE is a summary record submitted by a drug plan sponsor whenever a beneficiary fills a prescription under Medicare Part D. The PDE data are not the same as individual drug claim transactions, but are summary extracts using CMS-defined standard fields. The PDE record contains prescription drug cost and payment data that enables CMS to make payments to plans and otherwise administer the Part D benefit. Further information can be found by accessing the following link: http://www.cms.gov/Medicare/Prescription-Drug-Coverage/PrescriptionDrugCovGenIn/PartDData.html

Total Treatments displays the total number of treatments for Seroquel filled by your patients. A Seroquel treatment is defined as a patient visit to a pharmacy in which a Seroquel, Seroquel XR, or generic quetiapine prescription is filled. This data is shown in the graph on the first page of this letter. **30-Day Equivalent** reports the number of treatments adjusted by the days' supply of the drug dispensed.

Action

After reviewing this communication, you may be able to identify areas where your prescribing patterns could be modified, and we encourage you to share the trends that were identified with other clinicians. We hope you find this information helpful and that it will provide insight into your current and future prescribing practices.

If you would like to discuss this project or your data, or provide feedback on this analysis, please contact the NBI MEDIC at 1-877-7SafeRx (1-877-772-3379) or CMS at CPIMedicarePartD_Data@cms.hhs.gov. If you believe your prescriptions are being forged, please contact the NBI MEDIC at 1-877-7SafeRx
(1-877-772-3379).

If you would like more resources for detecting possible drug-seeking behavior on the part of your patients, please review the MLN Matters article on Prescription Drug Monitoring Programs (PDMPs) at https://www.cms.gov/Outreach-and-Education/Medicare-Learning-Network-MLN/MLNMattersArticles/Downloads/SE1250.pdf on the CMS website.

Thank you for your diligence and partnership with CMS in detecting, deterring and preventing fraud, waste and abuse in the Medicare Part C and Part D programs.

Attachment 4 (Followup Treatment Letter)

DEPARTMENT OF HEALTH & HUMAN SERVICES
CENTERS for MEDICARE & MEDICAID SERVICES
7500 Security Boulevard, Mail Stop AR-18-50
Baltimore, Maryland 21244-1850

CMS
CENTERS FOR MEDICARE & MEDICAID SERVICES
CENTER FOR PROGRAM INTEGRITY

Investigations & Audits Group

August 31, 2015

Pat Q. Provider MD (NPI: 1234567890)
1234 Main St
Columbia, MD 21045

Re: Your Seroquel prescribing is under review by the Center for Program Integrity.

Dear Dr. Provider,

We contacted you in April 2015 to communicate that your high prescribing of Seroquel (Seroquel, Seroquel XR, or generic quetiapine) was under review by the Center for Program Integrity. **We reached out because last year, you prescribed far more Seroquel than similar prescribers within your practice state.** In turn, you were flagged as a markedly unusual prescriber, subject to review by the Center for Program Integrity.

To assist in your monitoring efforts, the figure to the right displays our latest data on your prescribing of Seroquel in 2015 relative to other general care practitioners in your practice state - Maryland.

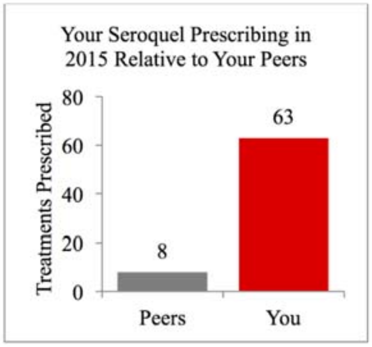

We recognize that some flagged practitioners have appropriate reasons for this pattern. However, we have seen that other practitioners may drift into prescribing patterns that would be considered medically unjustified or abusive. Abusive prescribing can lead to extensive audits and even revocation of Medicare billing privileges.

We hope that you will use this information to see if your high prescribing level is consistent with the latest standards of care, and we will continue to send you letters with our most recent information about your Seroquel prescribing. **We may contact you at a later date to ask what steps, if any, you have taken in response to our communications.**

Sincerely,

[redacted]

Investigations and Audits Group

Introduction

Prescribers and pharmacies have a frontline role in assisting the Centers for Medicare & Medicaid Services (CMS) to effectively manage Medicare resources and monitor prescribing practices. CMS and its partners acknowledge the daily challenges prescribers and pharmacies face in serving Medicare beneficiaries and the complexity of billing for prescription drugs.

The Office of Inspector General (OIG) released a study in June 2013 showing over 1 million individual prescribers ordered drugs paid by Medicare Part D in 2009. Prescribing patterns varied widely by type of prescriber. Over 700 general-care physicians had questionable prescribing patterns.[1] Although some of this prescribing may be appropriate, the OIG's study expressed the need to further scrutinize such questionable patterns.

Using a similar methodology to that used in the OIG study, CMS analyzed Seroquel, Seroquel XR, and generic quetiapine prescribing data for calendar year 2013 and 2014.[2] Based on this analysis, CMS has determined that the number and quantity of your treatments exceeded the established threshold (see box on right). The intent of this letter is to inform you of the extent of your potential outlier status, which reflects the Seroquel treatments attributed to your prescribing practice compared to your peers within your prescriber type (general care practitioner) and practice state (Maryland).

More Information

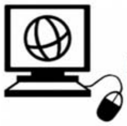

To learn more about Seroquel prescribing and to find details on our methodology, visit (this link is case sensitive):

http://go.cms.gov/CPImethodologyhr2

We hope you find this information helpful and that it will provide insights into your current and future prescribing practices. We also hope that you will use the information provided to see if your high prescribing level for Seroquel is appropriate for your patient population.

Your Results

Table 1: Your Recent Prescribing of Seroquel Treatments in Comparison to Your Peers

Categories	Total Treatments	30-Day Equivalent	Unique Beneficiary Count
Your Values in 2015 (to date*)	63	67.0	18
Average of Your Peers in 2015 (to date*)	7.9	10.5	3.3

* Preliminary data from PDE records received as of June, 2015.

[1] OIG, Prescribers with Questionable Patterns in Medicare Part D, OEI-02-09-00603, June 2013

[2] The prescribing data consists of Prescription Drug Event (PDE) records. Each PDE is a summary record submitted by a drug plan sponsor whenever a beneficiary fills a prescription under Medicare Part D. The PDE data are not the same as individual drug claim transactions, but are summary extracts using CMS-defined standard fields. The PDE record contains prescription drug cost and payment data that enables CMS to make payments to plans and otherwise administer the Part D benefit. Further information can be found by accessing the following link: http://www.cms.gov/Medicare/Prescription-Drug-Coverage/PrescriptionDrugCovGenIn/PartDData.html

This table reviews how your recent prescribing of Seroquel compares to your peers (other general care practitioners in Maryland).

Total Treatments displays the total number of treatments for Seroquel filled by your patients. A Seroquel treatment is defined as a patient visit to a pharmacy in which a Seroquel, Seroquel XR, or generic quetiapine prescription is filled. This data is shown in the graph on the first page of this letter. This treatment count also includes refills dispensed in this time period for earlier prescriptions.

30-Day Equivalent reports the number of treatments adjusted by the days' supply of the drug dispensed.

Unique Beneficiary Count shows the count of your Medicare patients who filled a treatment for Seroquel over this time period.

Table 2: Original Values Used to Determine Your Status as a Potential Outlier

Categories	Total Treatments	30-Day Equivalent	Unique Beneficiary Count
Your Original Values in 2014	211	222.7	33
Average of Your Peers in 2014	32.7	40.5	23.7

This table reviews the original data that led to your classification as a potential outlier, and was communicated to you already in our April 2015 memo. **Total Treatments, 30-Day Equivalent**, and **Unique Beneficiary Count** are defined as in Table 1.

Action

After reviewing this communication, you may be able to identify areas where your prescribing patterns could be modified, and we encourage you to share the trends that were identified with other clinicians. We hope you find this information helpful and that it will provide insight into your current and future prescribing practices.

If you would like to discuss this project or your data, or provide feedback on this analysis, please contact the NBI MEDIC at 1-877-7SafeRx (1-877-772-3379) or CMS at CPIMedicarePartD_Data@cms.hhs.gov. If you believe your prescriptions are being forged, please contact the NBI MEDIC at 1-877-7SafeRx (1-877-772-3379).

If you would like more resources for detecting possible drug-seeking behavior on the part of your patients, please review the MLN Matters article on Prescription Drug Monitoring Programs (PDMPs) at https://www.cms.gov/Outreach-and-Education/Medicare-Learning-Network-MLN/MLNMattersArticles/Downloads/SE1250.pdf on the CMS website.

Thank you for your diligence and partnership with CMS in detecting, deterring and preventing fraud, waste and abuse in the Medicare Part C and Part D programs.
