# Supplementary material for: Physician Antipsychotic Overprescribing Letters and Cognitive, Behavioral, and Physical Health Outcomes Among People With Dementia: A Secondary Analysis of a Randomized Clinical Trial
Source: JAMA Netw Open. 2024 Apr 25;7(4):e247604. doi: 10.1001/jamanetworkopen.2024.7604 (PMC11046341; doi:10.1001/jamanetworkopen.2024.7604)
Supplement: Supplement 2. — eFigure 1. CONSORT Flow Diagram of PCPs and Their Patients in Study eFigure 2. Effect of the Intervention on Nursing Home Assessment Outcomes in Patient Subgroups eFigure 3. Effect of the Intervention on Claims-Based Outcomes in Nursing Home Patient Subgroups eFigure 4. Effect of the Intervention on Claims-Based Outcomes in Community-Dwelling Patient Subgroups eTable 1. Effect of the Intervention During the First Six Quarters for Nursing Home Patients eTable 2. Effect on Other Prescribing Outcomes eTable 3. Effect on Alternative Measures of Cognitive, Behavioral, and Mental Health eTable 4. Effect on Additional Utilization Outcomes [file jamanetwopen-e247604-s002.pdf]

Supplemental Online Content

Harnisch M, Barnett ML, Coussens S, et al. Physician antipsychotic overprescribing letters and cognitive, behavioral, and physical health outcomes among people with dementia: a secondary analysis of a randomized clinical trial. *JAMA Netw Open*. 2024;7(4):e247604. doi:10.1001/jamanetworkopen.2024.7604

eFigure 1. CONSORT Flow Diagram of PCPs and Their Patients in Study
eFigure 2. Effect of the Intervention on Nursing Home Assessment Outcomes in Patient Subgroups
eFigure 3. Effect of the Intervention on Claims-Based Outcomes in Nursing Home Patient Subgroups
eFigure 4. Effect of the Intervention on Claims-Based Outcomes in Community-Dwelling Patient Subgroups
eTable 1. Effect of the Intervention During the First Six Quarters for Nursing Home Patients
eTable 2. Effect on Other Prescribing Outcomes
eTable 3. Effect on Alternative Measures of Cognitive, Behavioral, and Mental Health
eTable 4. Effect on Additional Utilization Outcomes

This supplemental material has been provided by the authors to give readers additional information about their work.

© 2024 Harnisch M et al. *JAMA Network Open.*

eFigure 1 – CONSORT Flow Diagram of PCPs and Their Patients in Study

Panel A: Nursing home patients

Panel B: Community-dwelling patients

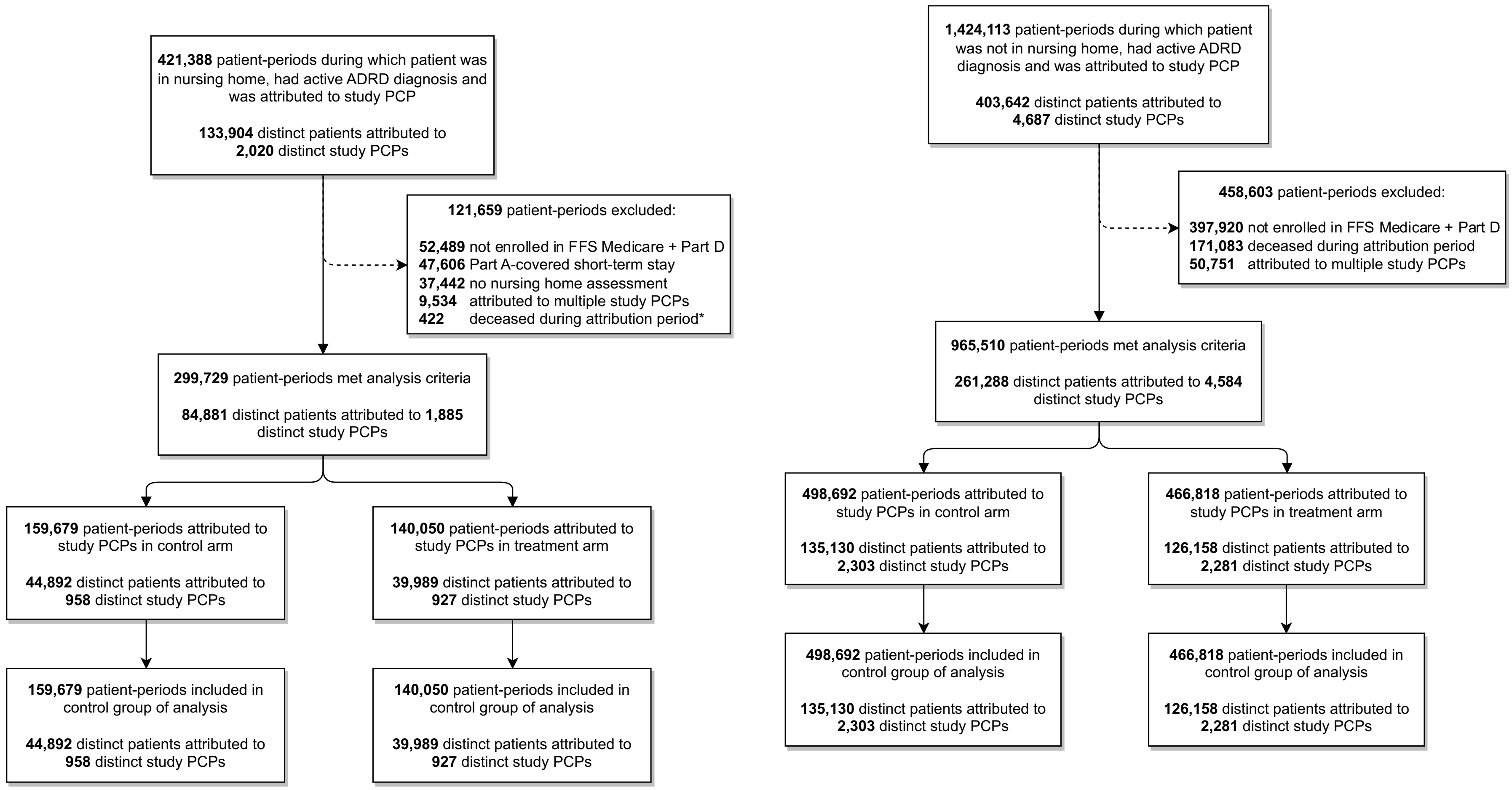

* In the nursing home sample, we exclude very few observations due to patient death during the attribution window. This number is low because patients only enter this sample if they reside in a nursing facility on the last day of the window. This critierion implicitly requires that they survive until the last day of the window

© 2024 Harnisch M et al. *JAMA Network Open.*

eFigure 2 – Effect of the Intervention on Nursing Home Assessment Outcomes in Patient Subgroups

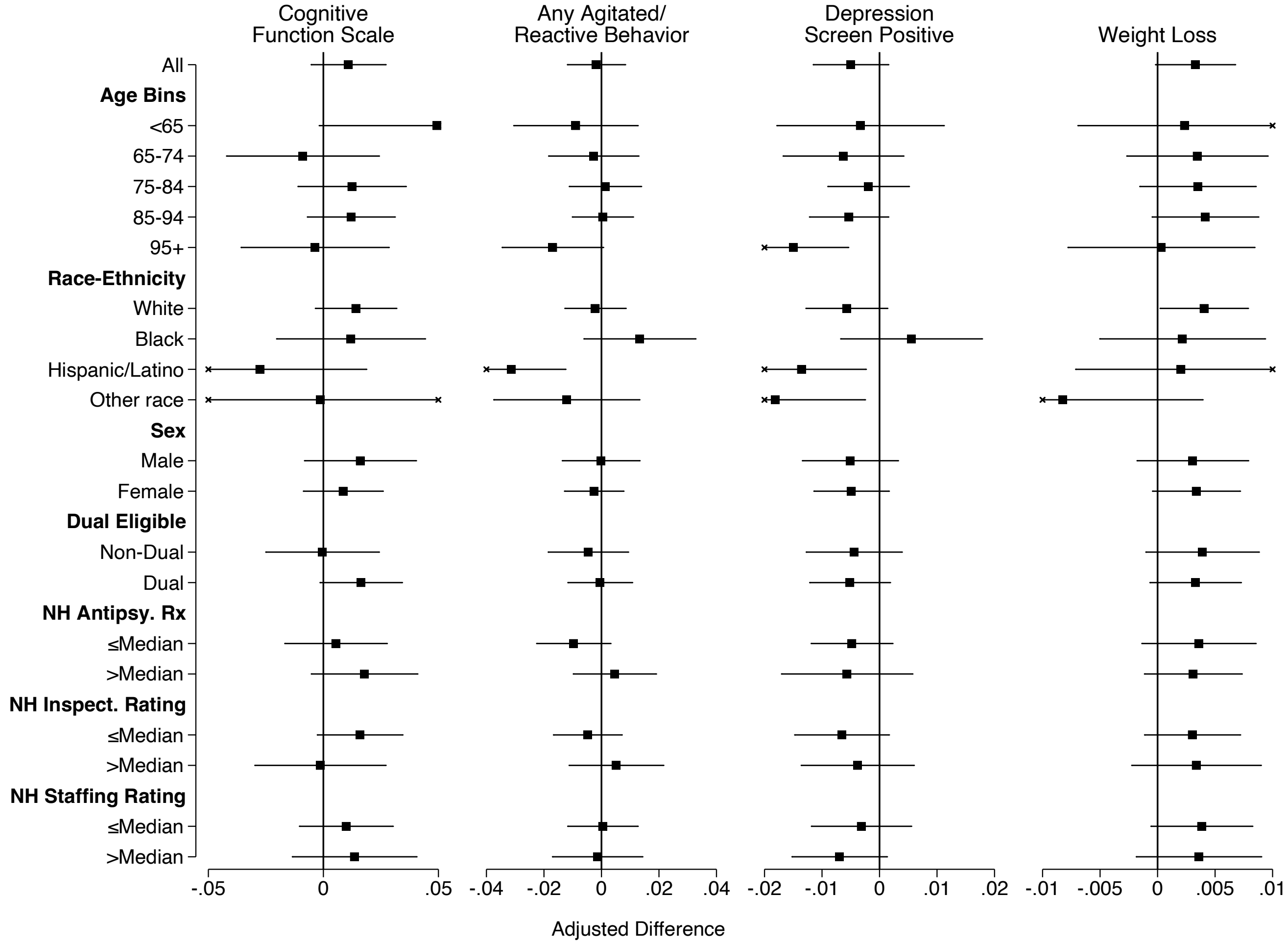

Notes: This figure visualizes effects of the intervention on selected indicators measured in nursing home assessments for patient subgroups. The first row shows the effect for the full sample (see Table 2). The final three subgroup analyses divide the sample based on indicators at the patient's nursing home: its antipsychotic prescribing rate, its safety inspection star rating, and its staffing rating, respectively. The Cognitive Function Scale ranges from 1=cognitively intact to 4=severely impaired. Any agitated/reactive behavior defined as Agitated and Reactive Behavior Scale > 0. Depression screen positive defined as Patient Health Questionnaire (PHQ-9) score ≥10. See Panel A of Figure 2 for subgroup sample sizes. Because assessment outcomes are occasionally missing, effective sample sizes are slightly smaller. Error bars show 95% confidence intervals. Bars truncated at the limits of the x-axis scale to improve visualization of effect differences. Truncation indicated by x at end of bar.
NH: nursing home.

© 2024 Harnisch M et al. *JAMA Network Open.*

eFigure 3 – Effect of the Intervention on Claims-Based Outcomes in Nursing Home Patient Subgroups

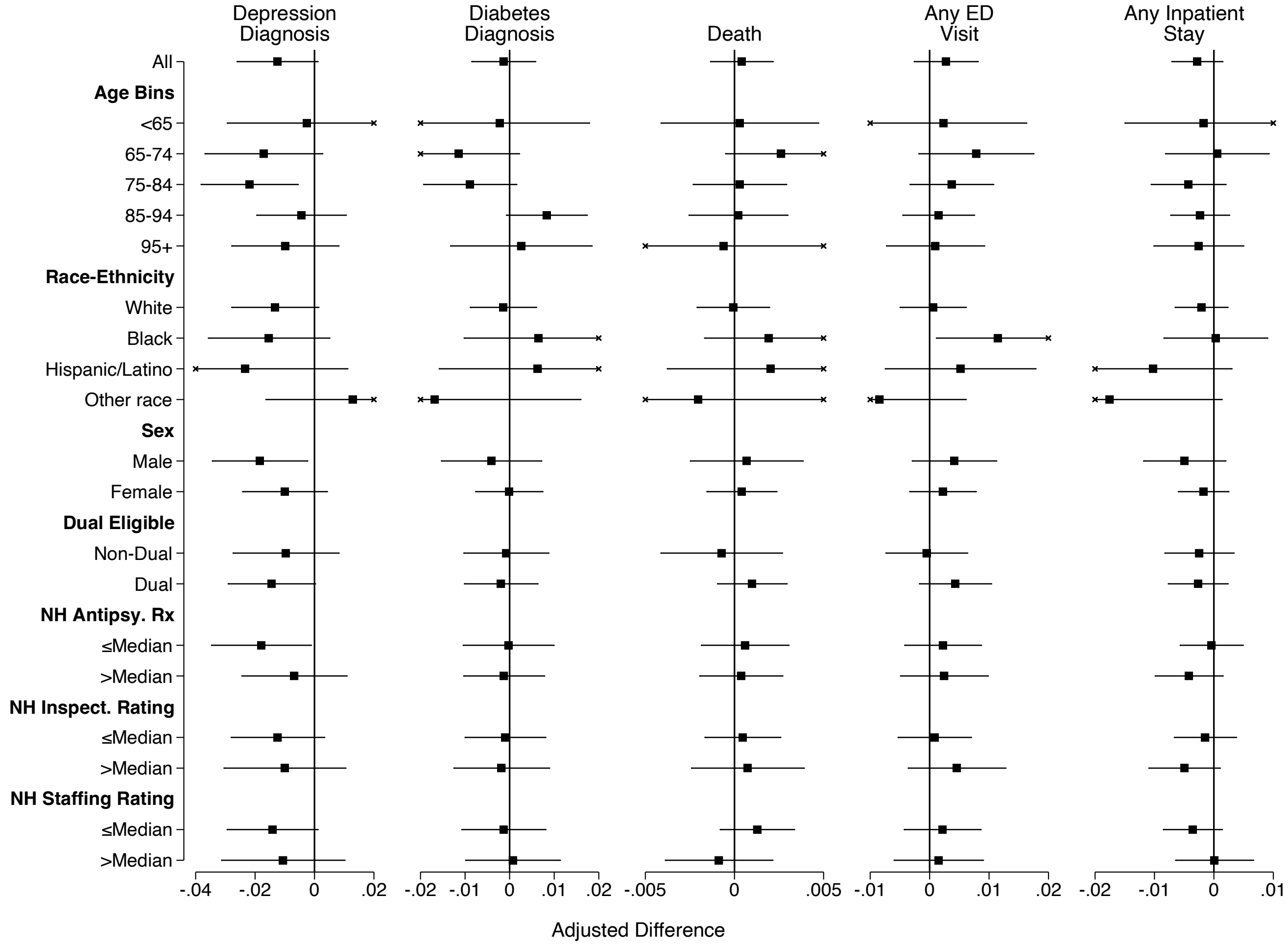

Notes: This figure visualizes effects of the intervention on selected indicators measured in claims for nursing home patient subgroups. The first row shows the effect for the full sample (see Table 2). The final three subgroup analyses divide the sample based on indicators at the patient's nursing home: its antipsychotic prescribing rate, its safety inspection star rating, and its staffing rating, respectively. See Panel A of Figure 2 for subgroup sample sizes. Error bars show 95% confidence intervals. Bars truncated at the limits of the x-axis scale to improve visualization of effect differences. Truncation indicated by x at end of bar.
NH: nursing home.

© 2024 Harnisch M et al. *JAMA Network Open.*

eFigure 4 – Effect of the Intervention on Claims-Based Outcomes in Community-Dwelling Patient Subgroups

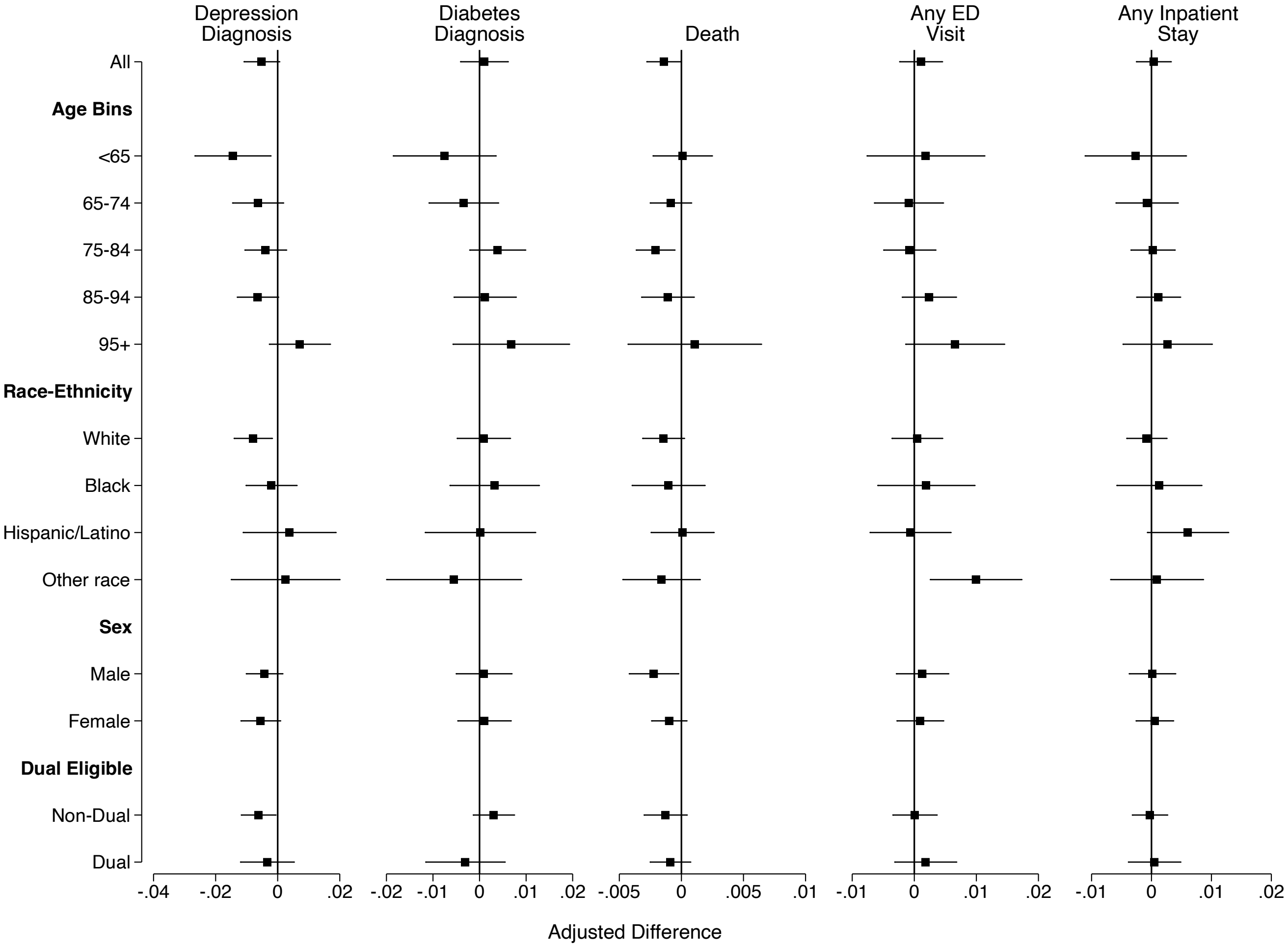

Notes: This figure visualizes effects of the intervention on selected indicators measured in claims for community-dwelling patient subgroups. The first row shows the effect for the full sample (see Table 2). See Panel B of Figure 2 for subgroup sample sizes. Error bars show 95% confidence intervals.

© 2024 Harnisch M et al. *JAMA Network Open.*

eTable 1 – Effect of the Intervention During the First Six Quarters for Nursing Home Patients

	Nursing home patients			
	Control mean	Adjusted difference (95% CI)		P value
**Prescribing**				
Quetiapine receipt				
Days	11.2	-1.05	(-1.59, -0.52)	<0.001
Days from attributed PCP	9.44	-0.83	(-1.36, -0.30)	0.00
Any receipt	11.29%	-0.87	(-1.36, -0.38)	<0.001
Milligrams	1,160	-79.7	(-144.0, -15.4)	0.02
Receipt of other antipsychotics				
Days	13.75	0.35	(-0.20, 0.90)	0.22
Any receipt	14.75%	0.51	(-0.01, 1.04)	0.05
Received other psych. medication	66.07%	0.24	(-0.51, 0.99)	0.54
**Cognitive and Behavioral Health**				
Cognitive function[a]	2.62	0.00	(-0.01, 0.02)	0.88
Agitated/ reactive behavior	19.41%	-0.17	(-1.04, 0.71)	0.71
Depression screen positive[b]	5.78%	-0.53	(-1.09, 0.04)	0.07
Depression diagnosis	29.94%	-1.50	(-2.77, -0.23)	0.02
**Metabolic Indicators and Diagnoses**				
Weight Loss	7.76%	0.51	(0.09, 0.92)	0.02
BMI	26.36	0.01	(-0.05, 0.08)	0.68
Diabetes diagnosis	34.76%	-0.39	(-1.03, 0.24)	0.23
Hyperlipidemia diagnosis	27.36%	0.05	(-1.18, 1.27)	0.94
Hypertension diagnosis	69.33%	-0.37	(-1.60, 0.87)	0.56
Hyperglycemia diagnosis	1.20%	-0.08	(-0.27, 0.11)	0.42
**Other Indicators of Adverse Events**				
Patient death	4.16%	-0.08	(-0.32, 0.15)	0.49
Any ED visit	10.64%	0.28	(-0.28, 0.84)	0.33
Any inpatient stay	11.03%	-0.30	(-0.79, 0.19)	0.23
Any use of restraints	2.18%	-0.08	(-0.41, 0.26)	0.66

* Exploratory analysis (not pre-specified). This table repeats the analyses of Table 2 but limits the sample to the first 6 quarters after study initiation. The number of observations is 129,594 patient-periods (50,316 distinct nursing home patients). Assessment outcomes are occasionally missing and effective sample sizes may be slightly smaller (minimum number of observations 126,013).

[a] Measured by the Cognitive Function Scale (CFS) ranging from 1=cognitively intact to 4=severely impaired.

[b] Defined as a Patient Health Questionnaire (PHQ-9) score ≥10.

PCP: primary care physician, ED: emergency department.

© 2024 Harnisch M et al. *JAMA Network Open.*

eTable 2 – Effect on Other Prescribing Outcomes

	Nursing home patients				Community-dwelling patients			
	Control mean	Adjusted difference (95% CI)		P value	Control mean	Adjusted difference (95% CI)		P value
**Quetiapine from Attributed PCP**								
Any receipt	9.06%	-0.70	(-1.21, -0.18)	0.01	7.73%	-1.46	(-1.76, -1.16)	<0.001
Milligrams[a]	890.7	-84.8	(-154.5, -15.0)	0.02	610.1	-108.0	(-141.9, -74.1)	<0.001
**All Antipsychotics**								
Days	23.31	-0.33	(-1.20, 0.54)	0.45	18.08	-1.38	(-1.81, -0.96)	<0.001
Any receipt	23.09%	-0.16	(-0.92, 0.60)	0.68	19.05%	-1.28	(-1.64, -0.92)	<0.001
Milligrams[a]	46.39	-0.09	(-2.08, 1.91)	0.93	36.00	-1.75	(-2.78, -0.72)	<0.001
Days in MDS[b]	1.53	-0.03	(-0.08, 0.03)	0.33				
**Other Psychoactive Medication**								
Antidepressants								
Days	58.43	-0.57	(-1.83, 0.69)	0.38	45.84	-0.10	(-0.74, 0.54)	0.76
Any receipt	55.14%	-0.42	(-1.33, 0.50)	0.37	43.74%	-0.08	(-0.55, 0.39)	0.73
Benzodiazepines								
Days	14.11	-0.08	(-0.62, 0.46)	0.77	13.98	0.21	(-0.08, 0.50)	0.16
Any receipt	20.98%	0.17	(-0.50, 0.83)	0.63	19.86%	0.27	(-0.08, 0.63)	0.13
Gabapentinoids								
Days	13.32	0.00	(-0.49, 0.49)	1.00	11.67	0.08	(-0.19, 0.35)	0.57
Any receipt	15.57%	-0.22	(-0.72, 0.28)	0.39	14.64%	0.07	(-0.25, 0.38)	0.68
Mood stabilizers								
Days	13.03	0.17	(-0.38, 0.71)	0.55	5.99	0.00	(-0.20, 0.19)	0.96
Any receipt	14.39%	0.19	(-0.37, 0.75)	0.51	6.60%	0.00	(-0.21, 0.22)	0.97
Non-benzodiazepine sedative-hypnotics								
Days	1.11	0.00	(-0.14, 0.15)	0.99	2.91	-0.02	(-0.15, 0.12)	0.82
Any receipt	1.65%	0.03	(-0.17, 0.22)	0.80	4.19%	-0.01	(-0.19, 0.17)	0.91

* All outcomes measured within 90-day periods and are based on claims with dates of service during the period. The number of observations is 299,729 patient-periods (84,881 distinct patients) for the nursing home sample and 965,510 patient-periods (261,288 distinct patients) for the community-dwelling sample.

[a] Risperidone milligram equivalents (see Supplement 1).

[b] Number of days patient received antipsychotics during the last 7 days according to nursing home assessment data.

© 2024 Harnisch M et al. *JAMA Network Open.*

eTable 3 – Effect on Alternative Measures of Cognitive, Behavioral, and Mental Health

	Nursing home patients			
	Control mean	Adjusted difference (95% CI)		P value
**Cognitive function**				
CFS = cognitively intact	19.25%	-0.53	(-1.23, 0.18)	0.15
CFS = mildly impaired	20.88%	0.37	(-0.29, 1.03)	0.27
CFS = moderately impaired	42.36%	-0.04	(-0.97, 0.89)	0.93
CFS = severely impaired	17.52%	0.33	(-0.34, 0.99)	0.34
**Agitated and reactive behavior**				
ARBS score	0.40	-0.02	(-0.05, 0.01)	0.16
**Delirium**				
Delirium indicator[a]	10.89%	0.12	(-0.81, 1.04)	0.80
**Depression**				
PHQ score	2.11	-0.10	(-0.21, 0.01)	0.09
PHQ > 0	46.97%	-0.82	(-2.50, 0.85)	0.34
PHQ = 0	55.27%	0.97	(-0.69, 2.62)	0.25
PHQ = 1-5	34.28%	-0.12	(-1.51, 1.26)	0.86
PHQ = 6-9	8.67%	-0.20	(-0.80, 0.41)	0.53

* All outcomes measured within 90-day periods and are based on assessments with target dates during the period. The number of observations ranges between 296,479 and 298,874 patient-periods (84,386 to 84,736 distinct patients).

[a] Analysis of delirium was not pre-specified.

CFS: Cognitive Function Scale, ARBS: Agitated and Reactive Behavior Scale, PHQ: patient health questionnaire.

© 2024 Harnisch M et al. *JAMA Network Open.*

eTable 4 – Effect on Additional Utilization Outcomes

	Nursing home patients				Community-dwelling patients			
	Control mean	Adjusted difference (95% CI)		P value	Control mean	Adjusted difference (95% CI)		P value
**Other Indicators of Adverse Events Continued**								
Any hospital visit for								
Cerebrovascular event	0.35%	0.00	(-0.05, 0.04)	0.96	0.57%	0.00	(-0.03, 0.04)	0.79
Mental health principal diagnosis	0.48%	-0.02	(-0.10, 0.06)	0.57	0.46%	0.01	(-0.03, 0.06)	0.55
**Other Indicators of Health Care Use**								
Any visit with								
Attributed PCP	90.42%	0.25	(-0.84, 1.33)	0.65	73.62%	-0.28	(-0.93, 0.37)	0.40
Primary care provider	93.98%	0.21	(-0.60, 1.02)	0.60	83.31%	-0.12	(-0.56, 0.33)	0.61
Geriatrician	10.78%	-0.12	(-0.60, 0.36)	0.62	8.45%	-0.11	(-0.45, 0.24)	0.54
Psychiatrist	12.26%	0.36	(-0.97, 1.69)	0.60	4.81%	0.12	(-0.22, 0.45)	0.50
Psychologist	5.75%	-1.13	(-1.87, -0.39)	0.00	0.99%	-0.08	(-0.19, 0.03)	0.14
Therapy receipt	1.01%	-0.03	(-0.26, 0.19)	0.76				

* All outcomes measured within 90-day periods and are based on claims and assessments with dates of service or target dates during the period. The number of observations is 299,729 patient-periods (84,881 distinct patients) for the nursing home sample and 965,510 patient-periods (261,288 distinct patients) for the community-dwelling sample. The number of observations for therapy receipt is lower (94,529 patient-periods and 55,089 distinct patients) as this outcome is only reported annually in the MDS and not during each quarterly assessment.
PCP: primary care physician.

© 2024 Harnisch M et al. *JAMA Network Open.*
