# Supplementary material for: Physician Antipsychotic Overprescribing Letters and Cognitive, Behavioral, and Physical Health Outcomes Among People With Dementia: A Secondary Analysis of a Randomized Clinical Trial
Source: JAMA Netw Open. 2024 Apr 25;7(4):e247604. doi: 10.1001/jamanetworkopen.2024.7604 (PMC11046341; doi:10.1001/jamanetworkopen.2024.7604)
Supplement: Supplement 3. — Data Sharing Statement [file jamanetwopen-e247604-s003.pdf]

Data Sharing Statement

Harnisch. Physician Antipsychotic Overprescribing Letters and Cognitive, Behavioral, and Physical Health Outcomes Among People With Dementia. *JAMA Netw Open*. Published April 25, 2024. doi:10.1001/jamanetworkopen.2024.7604

Data
Data available: No

Additional Information
Explanation for why data not available: This study uses Medicare research identifiable files (RIFs). Due to the terms of the RIF data use agreement, we are not able to make individual patient data available.
